# Involvement of mTOR-autophagy in the selection of primitive mesenchymal stem cells in chitosan film 3-dimensional culture

**DOI:** 10.1038/s41598-017-10708-0

**Published:** 2017-08-31

**Authors:** Hsiao-Ying Chiu, Yeou-Guang Tsay, Shih-Chieh Hung

**Affiliations:** 1 Department of Medical Research, Taipei Veterans General Hospital, Taipei, Taiwan; 2 Department of Orthopaedics & Traumatology, Taipei Veterans General Hospital, Taipei, Taiwan; 3 Institute of Biomedical Sciences, Academia Sinica, Taipei, Taiwan; 4 Institute of Biochemistry and Molecular Biology, National Yang-Ming University, Taipei, Taiwan; 5 Proteomics Research Center, National Yang-Ming University, Taipei, Taiwan; 6 Department of Orthopaedics, and Integrative Stem Cell Center, China Medical University Hospital, Taichung, Taiwan; 7 Institute of New Drug Development, Biomedical Sciences, China Medical University, Taichung, Taiwan

## Abstract

Mesenchymal stem cells (MSCs) in conventional monolayer culture are heterogeneous and contain a significant portion of senescent cells. MSCs cultured on chitosan film form 3-dimenional spheres, increase in stemness and differentiation capability; however, the underlying mechanisms remain elusive. We first demonstrate chitosan film culture induces apoptosis at 2 days, with specificity in late senescent cells. Especially in senescent cells, chitosan film culture activates mTOR, which activates S6K/S6/4E-BP1 to enhance fibronection synthesis and peripheral dead cell attachment, and phosphorylates ULK1 at S757 to further inactivate ULK1, LC3A and autophagy, thereby inducing apoptosis. Combination of chitosan film culture with mTOR inhibition prevents peripheral dead cell attachment, thereby further increasing pluripotent gene expression, *in vitro* osteogenesis and *in vivo* bone formation. These data successfully figure out the role of mTOR signaling in chitosan film culture and develop a method by combination of rapamycin treatment for promoting stemness and differentiation capability in MSCs.

## Introduction

Human mesenchymal stem cells (MSCs), with the capability for self-renewal and differentiation into various mesenchymal and non-mesenchymal tissues^1, 2^. The usefulness of MSCs for the treatment of musculoskeletal disorder, including osteogenesis imperfecta^3^ and tissue engineering in orthopaedics^4^ has been explored and MSCs are currently under evaluation in clinics. However, various isolation and expansion techniques cause a remarkable difference in their proliferation capacity and differentiation potentials^5^. Furthermore, clinical applications of MSCs require a large number of expanded cells. However, many studies have consistently reported expanded MSCs are heterogeneous and contain a significant portion of senescent cells^6^. Moreover, MSCs often lose their stemness and multi-differentiation abilities when cultured in conventional two-dimensional (2D) systems. Thus, the development of novel culture methods for expanding homogenous and non-senescent MSCs without the loss of proliferation, stemness and multi-differentiation abilities attracts a great interest in the research field.

Previous studies have identified the effects of biomaterials, such as type I collagen on microsphere formation and stemness maintenance in MSCs^7^. Recently, many studies have also studied the effects of chitosan film or membrane on the morphology, stemness and multi-differentiation abilities of MSCs. It has been demonstrated that MSCs cultured on chitosan film form spheres. Additionally, the expression of stemness marker genes, including Oct4, Nanog and Sox2, increased significantly when MSCs were cultured using chitosan film compared with 2D monolayer culture systems^8, 9^. More importantly, culture on chitosan film resulted in an increased differentiation potential of MSCs into mesenchymal lineages, such as osteoblasts^8, 9^, and non-mesenchymal lineages, such as nerve cells^10^. However, the underlying mechanisms that MSCs cultured on chitosan film mediated to form sphere and increase in the stemness and differentiation abilities remain elusive.

The mammalian target of rapamycin (mTOR) kinase is present in two functionally and structurally distinct multiprotein complexes termed TOR complex 1 (TORC1, consisting of mTOR, Raptor and mLST8) and TOR complex 2 (TORC2, consisting of mTOR, Rictorm mSIN1, Rictor and mLST8)^11, 12^, the former is rapamycin-sensitive, while the latter is not directly inhibited by rapamycin^13^. mTORC1 has been known for controlling many cellular processes, including protein synthesis, ribosome biogenesis, nutrient transport and autophagy. The two best-characterized down-stream substrates of mTORC1 are S6 kinase (S6K) and 4E binding protein 1 (4E-BP1), via which mTORC1 controls protein synthesis^14^. Emerging evidence has shown that mTOR may modify cell proliferation and differentiation of many cell types, including MSCs^15^.

In the present study, we first showed that MSCs when cultured on chitosan film for 7 days, similar to those reported previously^8, 9^, formed 3-dimenional (3D) spheres, increased in the expression of Oct4, Nanog and Sox2, and enhanced osteogenic differentiation potential upon re-plate in monolayer culture and induction for osteogenesis. However, we also found when cultured on chitosan film for 2 or 3 days, MSCs underwent significant apoptosis, which inversely correlated with the primitive status of MSCs. Moreover, western blotting and immunostaining analyses revealed MSCs increased in the activation of mTOR and its downstream molecule S6K, a protein synthesis signaling. Interestingly, we also found autophagy signaling molecules, such as ULK1, LC3, which have been reported suppressed by mTOR, were suppressed upon culture on chitosan film. Through inhibition of mTOR by a specific inhibitor or shRNAs, we further demonstrate mTOR activation during chitosan film culture affects fibronectin synthesis and apoptosis, thereby having the ability to form sphere and increase in the stemness and osteogenic differentiation abilities through ablation of senescent cells. These data successfully figure out the relationship between mTOR signaling and the chitosan film culture-mediated sphere formation, and increases in the expression of pluripotent genes, replicative and osteogenic differentiation potential.

## Results

### Chitosan film culture induces sphere formation and promotes pluripotent gene expression, proliferation and osteogenic differentiation potential of MSCs

We first isolated MSCs from three individuals, which are positive for CD29, CD44, CD73, CD90 and CD105, but negative for CD11b, CD34, CD45, CD79a and HLA-DR, and possess the ability to differentiate into osteoblasts, chondrocytes and adipocytes (Supplementary Figure 1). To examine whether chitosan film culture increases sphere formation, these MSCs were seeded in dishes coated without (monolayer) or with chitosan (chitosan film) and the ability of cells to form spheres was assayed. As previously reported^8, 9^, chitosan film culture increased sphere formation of MSCs at different culture densities (Supplementary Figure 2a and Supplementary Figure 3). Moreover, the size of spheres correlated with the seeding density and increased along with the increase of culture period (Supplementary Figure 3). These data suggest MSCs form spheres on chitosan film. To examine the effects of chitosan film culture on stem cell properties of MSCs, the expression of pluripotent genes, the percentage of senescent cells, the proliferation ability, and osteogenic differentiation potential were compared between MSCs grown at the density of 2.5 × 10^4^/cm^2^ in monolayer and chitosan film culture for 7 days. MSCs recovered from chitosan film culture increased in the expression of Oct4, Nanog and Sox2, compared to MSCs recovered from monolayer culture (Supplementary Figure 2b). For analysing the percentage of senescent cells, MSCs recovered from monolayer and chitosan film culture were seeded in monolayer, followed by assaying the senescence-associated β-galactosidase activity (SA–β-Gal) at 24 hr. MSCs recovered from chitosan film culture showed a decreased percentage of cells positive for SA–β-Gal (4.9±0.9%), compared to MSCs recovered from monolayer culture (12.5±1.7%) (Supplementary Figure 2c). Tracking of cell division following carboxyfluorescein diacetate succinimidyl ester (CFSE) labeling and reseeding in monolayer revealed MSCs recovered from chitosan film culture showed increased replication and replicated as a more homogenous population, while MSCs recovered from monolayer culture showed decreased proliferation and failed to replicate consistently (Supplementary Figure 2d). More importantly, MSCs recovered from chitosan film culture also showed increased osteogenic potential when reseeded and induced with osteogenic differentiation medium for 14 days when compared to MSCs recovered from monolayer (Supplementary Figure 2e). The data suggest chitosan film culture promotes pluripotent gene expression, inhibits senescence, and enhances proliferation and osteogenic differentiation potential of MSCs.

### Chitosan film culture induces apoptosis in MSCs via caspase 9 and 3 dependent pathway

**Figure 1:**
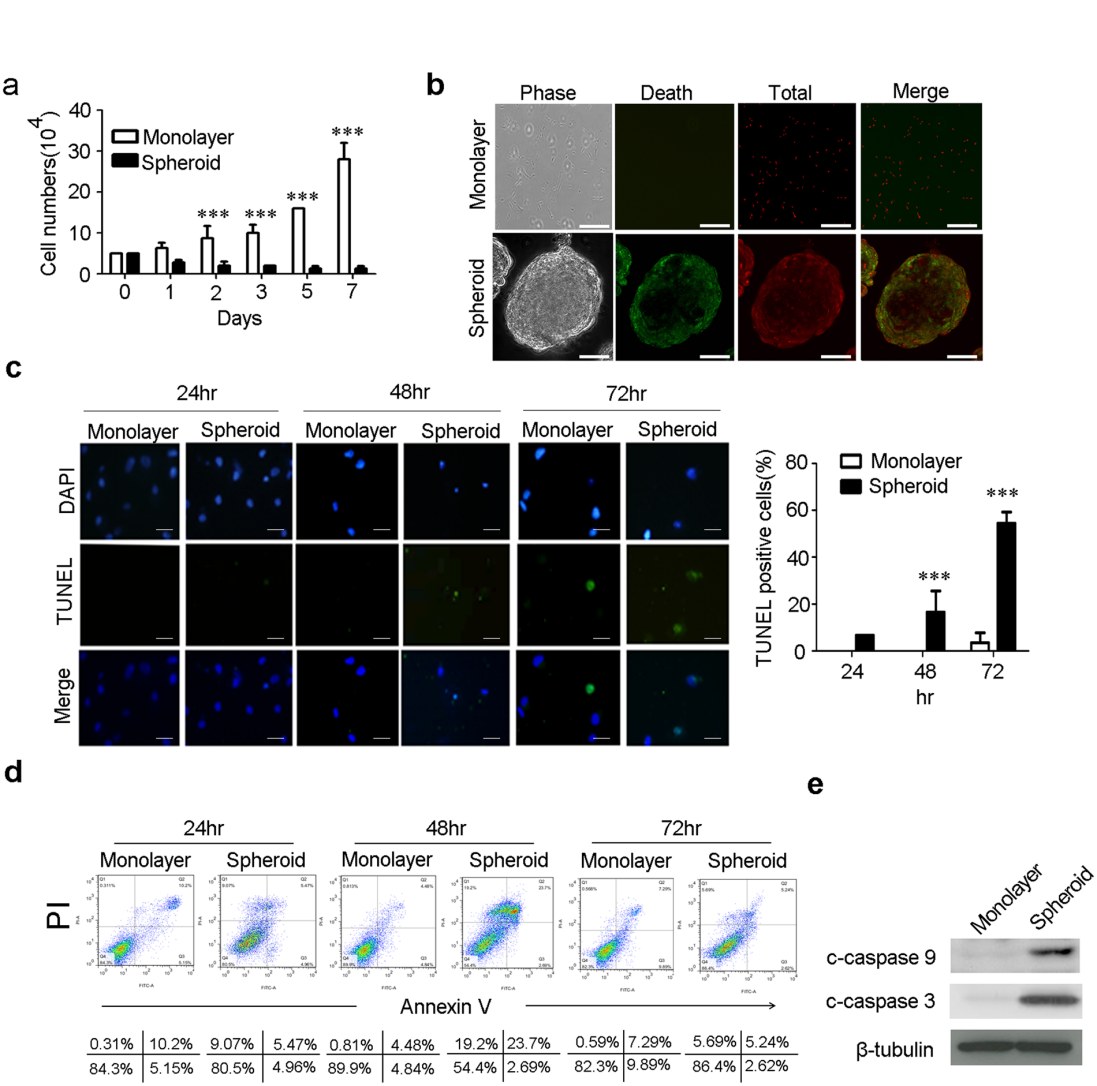
The spheroid formation on chitosan film is associated with an increase in Apoptosis. MSCs were seeded at 2.5 × 10^4^/cm^2^ without (Monolayer) or with chitosan coating (Spheroid), followed by calculation of cell numbers (**a**), Live/Dead staining (48 hr; Scale bar = 50 μM) (**b**), TUNEL assay (Scale bar = 10 μM) (**c**), and Annexin V/PI assay (**d**) at indicated time points. (**e**) Whole-cell lysates at 48 hr were analyzed by western blotting with specific antibodies against c-caspase-9 and c-caspase 3. β-tubulin was used as a loading control. The results are expressed as the mean ± standard deviation of three independent experiments, which is representative of MSCs from two individuals. ***p < 0.005 compared with monolayer cells.

Since MSCs are heterogeneous and more primitive stem cells are prone to survive in sphere culture^16^, we therefore doubted whether the increased pluripotent gene expression and enhanced stem cell properties were attributed to the selection of more primitive MSCs from the heterogeneous population. MSCs were seeded in dishes coated without or with chitosan and assayed for cell number changes and apoptosis for up to 7 days. Cell numbers in monolayer culture increased exponentially after day 2, while the cell numbers in chitosan film culture decreased along with culture period up to 3 days, then maintained almost the same after day 3 (Fig. 1a). These data suggest MSCs grow in monolayer culture, while undergo cell loss at early stage of chitosan sphere culture. Because most of the cell loss in chitosan film culture happened from day 1 to day 3, we then analyzed cell apoptosis and cell death at this period. Live/Dead staining revealed chitosan film culture increased cell death with prominent cell death occurring in the periphery of the sphere (Fig. 1b). TUNEL assay demonstrated chitosan film culture significantly increased apoptosis rate at 2 and 3 days (Fig. 1c). Annexin V/PI flow cytometric analysis also revealed chitosan film culture increased apoptosis and necrosis rates with peak at day 2 to day 3 and returning to the level of day 1 at day 7 (Fig. 1d and Supplementary Figure S4). Moreover, chitosan film culture increased in the cleavage of caspase 9 and 3 (Fig. 1e), and inhibitors of caspase 9 and 3 increased cell survival in chitosan film culture (Supplementary Figure S5), suggesting chitosan film culture induces apoptosis in MSCs through caspase 9 and caspase 3-dependent pathways.

### Early-passage MSCs were more resistant to chitosan film culture-induced apoptosis than late-passage MSCs

**Figure 2:**
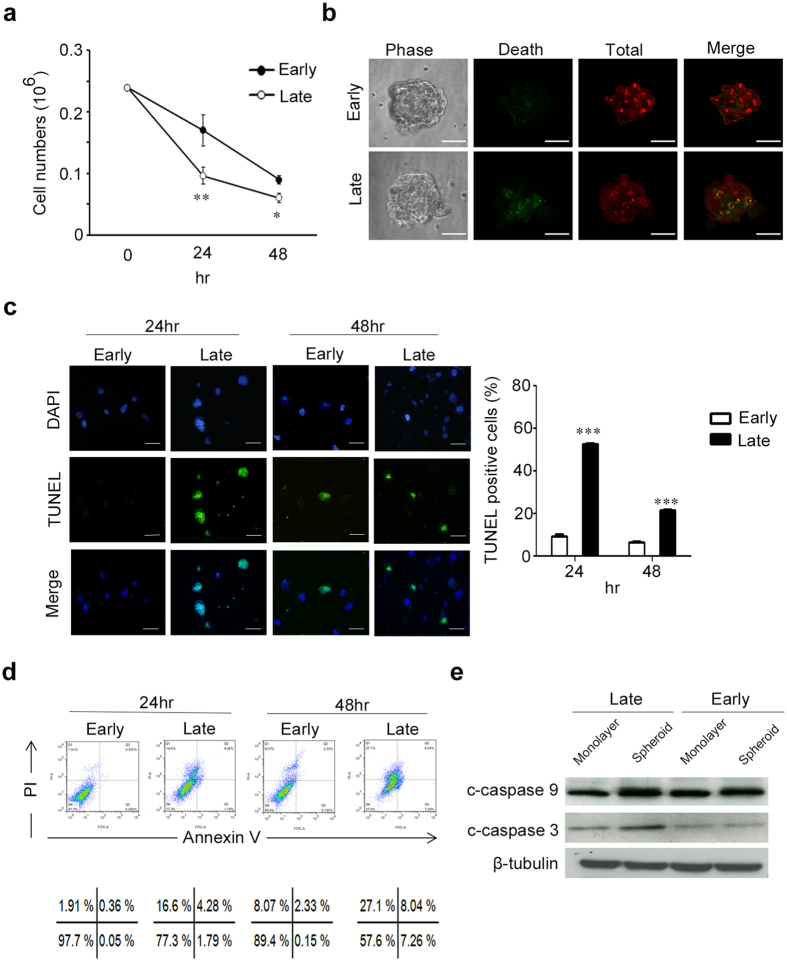
Early-passage MSCs increase in survival compared with late-passage MSCs on chitosan film. MSCs of early and late passages were seeded in dishes coated with chitosan, followed by calculation of cell numbers (**a**), Live/Dead staining (48 hr; Scale bar = 50 μM) (**b**), TUNEL assay (Scale bar = 10 μM) (**c**), and Annexin V/PI assay (**d**) at indicated time points. (**e**) Whole-cell lysates at 48 hr were analyzed by western blotting with specific antibodies against c-caspase-9 and c-caspase 3. β-tubulin was used as a loading control. The results are expressed as the mean ± standard deviation of three independent experiments, which is representative of MSCs from two individuals. *p < 0.05, **p < 0.01 and ***p < 0.005 compared with monolayer cells.

To demonstrate chitosan film culture selectively induced more apoptosis in late senescent MSCs than in early primitive MSCs, we compared the survival ratio on chitosan film between early-passage (passage 1–2) and late-passage (passage 6–7) MSCs that increased in SA–β-Gal staining (Supplementary Figure S6). Analysis of cell number by trypan blue exclusion method showed late-passage MSCs decreased in live cell number compared to early-passage MSCs (Fig. 2a). Live/dead staining also revealed chitosan film culture induced more cell death in late-passage MSCs compared to early-passage MSCs (Fig. 2b). Moreover, late-passage MSCs increased in apoptosis rate on chitosan film compared to early-passage MSCs as demonstrated by TUNEL assay (Fig. 2c), Annexin V/PI flow cytometric analysis (Fig. 2d) and western blotting for the cleavage of caspase 9 and caspase 3 (Fig. 2e). These data together suggest early-passage MSCs increase in survival upon culturing on chitosan film compared to late-passage MSCs.

### Chitosan film culture has higher levels of phospho-mTOR, phospho-S6K and phospho-S6 compared to monolayer culture

**Figure 3:**
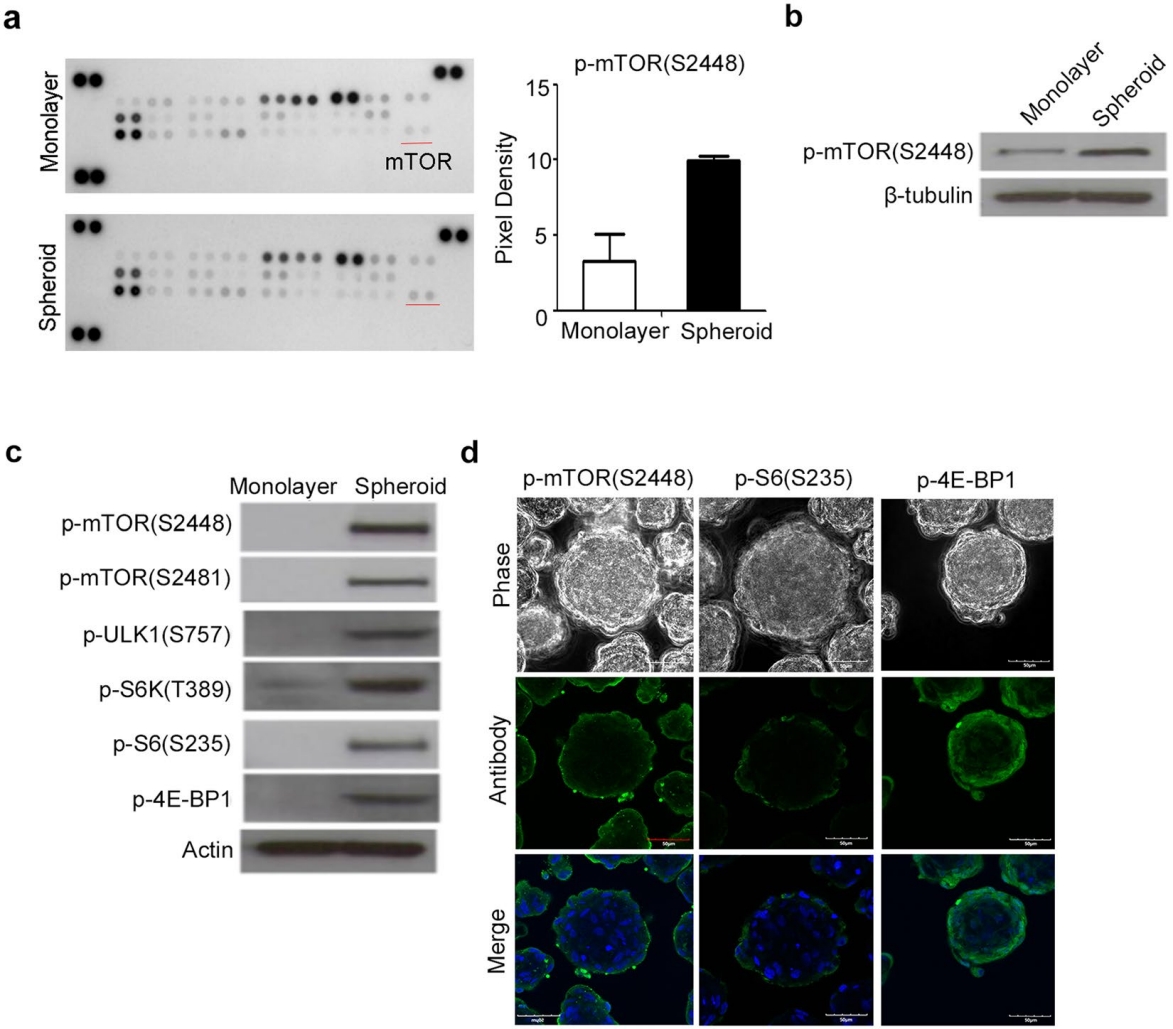
Activation of the mTOR signaling pathway during spheroid formation on chitosan film. (**a**, Left panel) After 48 hr of seeding on monolayer or with chitosan coating (Spheroid), cell lysates were analyzed using the Human Phospho-MAPK Array kit. (**a**, Right panel) the spots of phospho-mTOR (S2448) were analyzed by an image analysis system. (**b** and **c**) Whole-cell lysates were analyzed by western blotting with specific antibodies against indicated molecules. β-tubulin and actin were used as the loading control. (**d**) Immunostaining of p-mTOR (S2448), p-S6 (S235), and p-4EBP1 in 3D spheres. DAPI merged images are shown in the low panel. Scale bar = 50 μM. The results are expressed as the mean ± standard deviation of three independent experiments, which is representative of MSCs from two individuals. **p < 0.01 compared with monolayer cells.

To gain insight into the underlying mechanism or signaling pathways that chitosan film culture mediated to induce sphere formation and select more primitive cells for promoting stemness on MSCs, a MAPK array was used to identify the pathways activated upon culturing cells on chitosan film (Fig. 3a). Comparing to the monolayer culture, the mTOR was one of the most activated signaling molecules at 48 hr after seeding on chitosan film. Western blot analysis was performed to confirm the activation of mTOR (phosphorylation at S2448 and S2481) upon culturing MSCs on chitosan film (Fig. 3b,c). The activation of mTOR upon culturing on chitosan film has also been demonstrated in MSCs from two other individuals. Consistently, western blot analysis also demonstrated the increase of phosphorylation levels of mTOR downstream singling molecules, such as S6K, S6, and 4E-BP1 (Fig. 3c). Immunofluorescence further revealed the phosphorylated forms of mTOR, S6K, and 4E-BP1 were restricted to the periphery of the spheres (Fig. 3d). Together, these data suggest the topographic difference in the activation of mTOR and its downstream signaling molecules in the spheres.

### The effects of mTORC1 inhibitor on sphere formation, stemness maintenance of MSCs on chitosan film

**Figure 4:**
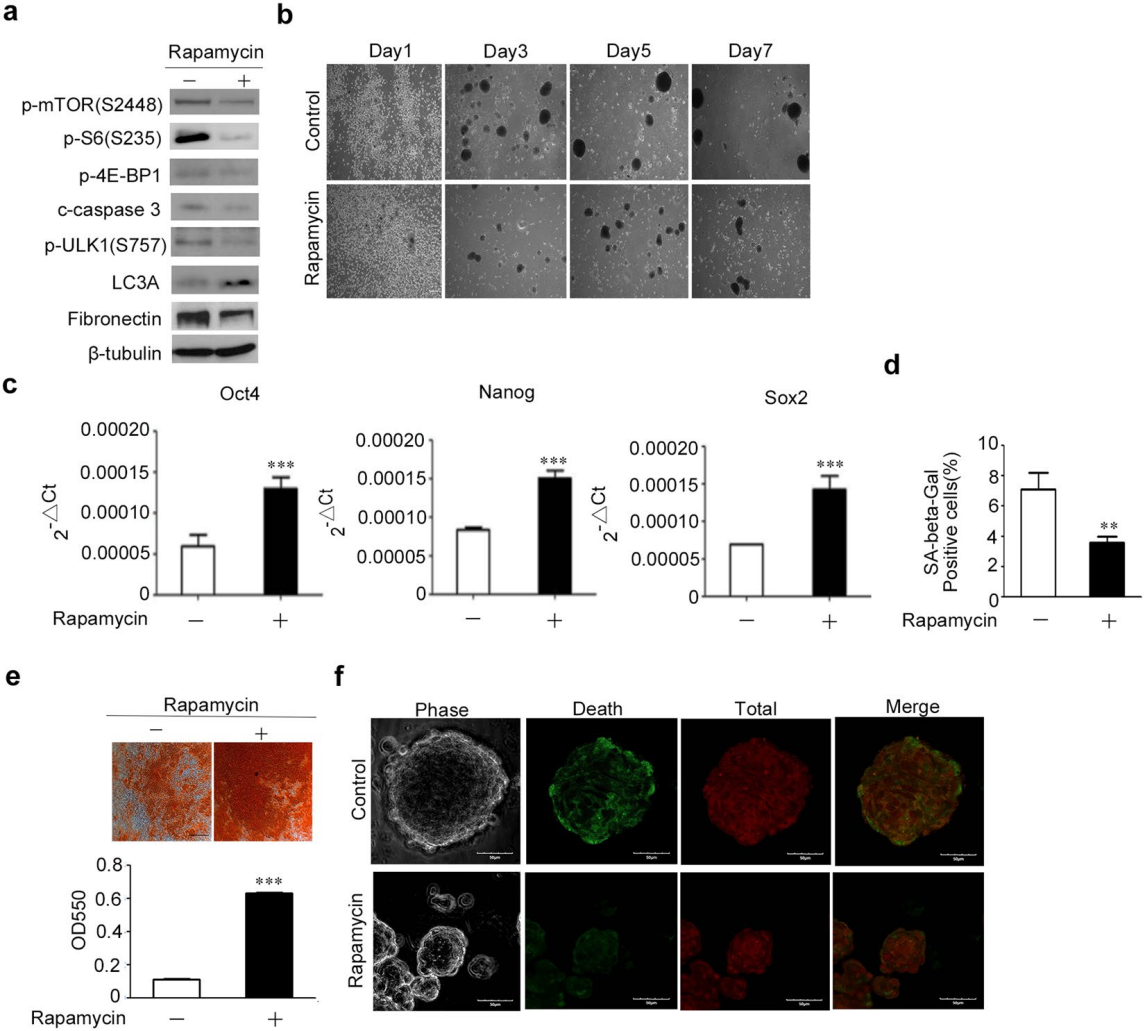
Rapamycin treatment in chitosan film culture decreases sphere formation and enhances stem cell properties in MSCs. MSCs were seeded at 2.5 × 10^4^/cm^2^ in dishes coated with chitosan in the absence or presence of rapamycin. (**a**) After 48 hr of seeding, cell lysates were assayed with western blotting analysis. (**b**) Representative morphologies of indicated time points are shown. Scale bar = 100 μM. (**c**) After 7 days of seeding, the mRNA levels of Oct4, Nanog and SOX2 were analyzed by quantitative RT-PCR. (**d**) After 7 days of seeding, cells were reseeded and assayed for the senescence-associated β-galactosidase (SA-β-Gal) staining. (**e**) After 7 days of seeding, cells were reseeded and induced for osteogenic differentiation for 14 days, followed by alizarin red S staining (Upper panel) and optical density measurement of extracted dyes at 550 nm (Lower panel). Scale bar = 100 μM. (**f**) MSCs treated without (control) or with rapamycin for 2 days were assayed by Live/Dead staining. Cell tracker stained cells appear red, and dead cells appear green. All images were intensity matched and were taken at the base of the cell using a microscope (Scale bar = 50 μM). The results are expressed as the mean ± standard deviation of three independent experiments, which is representative of MSCs from two individuals. **p < 0.01 and ***p < 0.005 compared with control.

mTOR forms two types of complexes, mTORC1 and mTORC2, both of which were activated by the phosphorylation of mTOR and only mTORC1 is inhibited by rapamycin^17^. We then examined whether the mTORC1 pathway is involved in sphere formation, selection of more primitive MSCs on chitosan film by blocking mTOR with rapamycin. Upon treatment with rapamycin, MSCs decreased in the phosphorylation levels of mTOR, and its downstream molecules, such as S6K-S6, 4E-BP1, and cleaved caspase 3 (Fig. 4a and Supplementary Figure S7a). These data suggest mTORC1 was activated in MSCs upon culturing on chitosan film. Interestingly, spheres formed by MSCs in the presence of rapamycin decreased in size compared to that formed in the absence of rapamycin (Fig. 4b and Supplementary Figure S7b). More importantly, quantitative RT-PCR revealed that rapamycin treatment increased the mRNA levels of Oct4, Nanog and Sox2, suggesting mTORC1 inhibition further upregulated pluripotent gene expression that has already upregulated by chitosan film culture (Fig. 4c). SA–β-Gal staining (Fig. 4d) also revealed the percentage of cells positive for SA–β-Gal activity was decreased in cells treated with rapamycin (3.6 ± 0.4%) compared to cells without treatment with rapamycin (7.1 ± 1.1%). Moreover, MSCs recovered from spheres formed in the presence of rapamycin, increased in osteogenesis when compared to MSCs recovered from spheres formed in the absence of rapamycin (Fig. 4e). Interestingly, we found rapamycin treatment reduced the dead cell numbers that were previously observed in the periphery of the sphere (Fig. 4f). Together, these data suggest inhibition of mTORC1 by rapamycin treatment decreases sphere size, upregulates MSC stem cell properties and reduces the dead cells in the periphery of sphere.

### Effects of mTOR knockdown on sphere formation, stemness maintenance of MSCs on chitosan film

**Figure 5:**
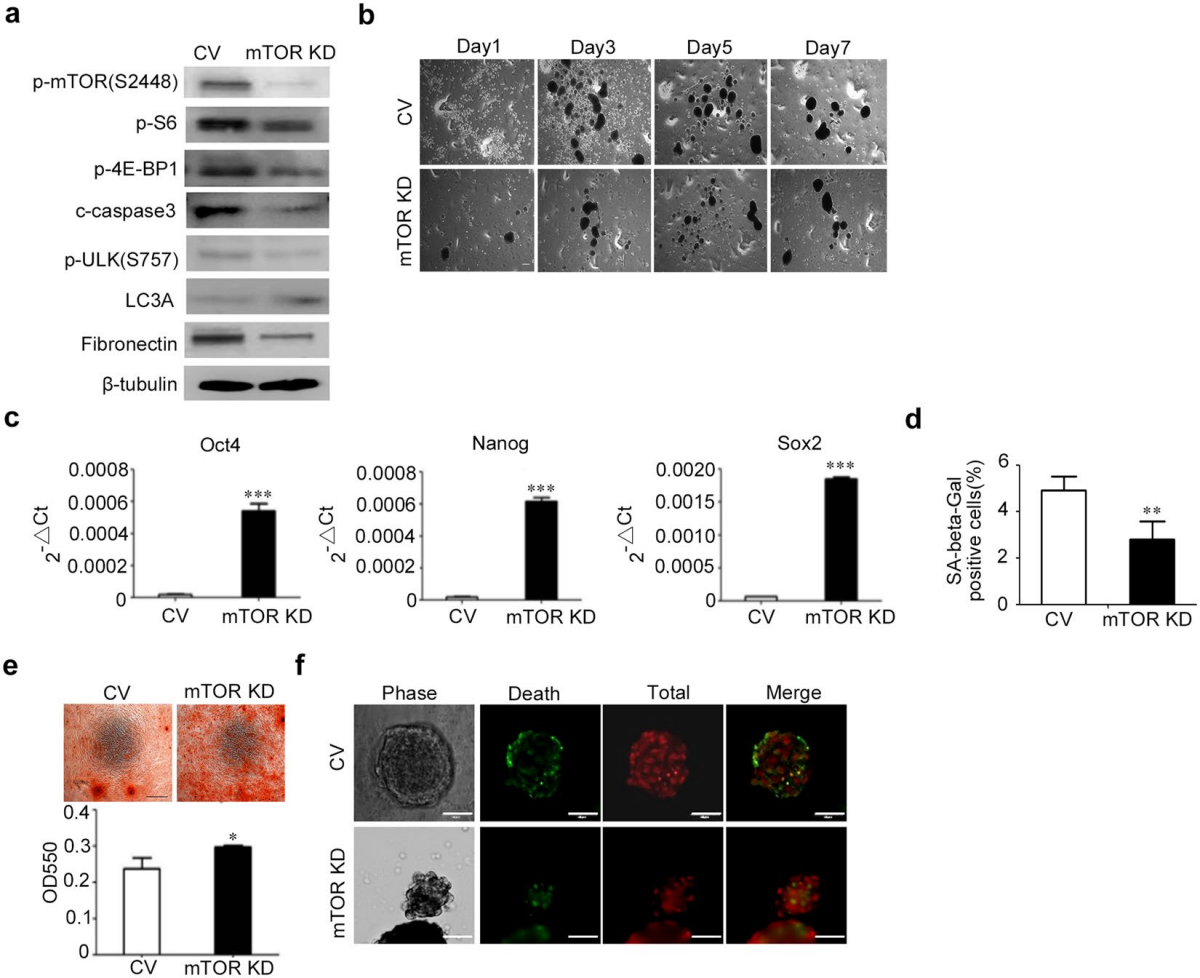
Knockdown of mTOR in chitosan film culture decreases sphere formation and enhances stem cell poperties in MSCs. (**a**) MSCs were transfected with shRNAs against mTOR (mTOR KD) and seeded in dishes coated with chitosan at 2.5 × 10^4^/cm^2^. Transfection with non-specific shRNAs (control vector, CV) was used as a control. After 48 hr of seeding, cell lysates were assayed using western blotting analysis. β-tubulin was used as a loading control. (**b**) Representative bright field micrographs at indicated time points show the morphological features. Scale bar = 100 μM. (**b**) After 7 days of seeding, the mRNA levels of Oct4, Nanog and SOX2 were analyzed by quantitative RT-PCR. (**d**) After 7 days of seeding, cells were reseeded and assayed for the senescence-associated β-galactosidase (SA-β-Gal) staining. (**e**) After 7 days of seeding, cells were reseeded and induced for osteogenic differentiation for 14 days, followed by alizarin red S staining (Upper panel) and optical density measurement of extracted dyes at 550 nm (Lower panel). Scale bar = 100 μM. (**f**) After 48 hr of seeding, MSCs were assayed for Live/Dead staining (Scale bar = 50 μM). The results are expressed as the mean ± standard deviation of three independent experiments, which is representative of MSCs from two individuals. **p < 0.01, ***p < 0.005 compared with control.

We then examined whether mTOR pathways is involved in sphere formation, stemness maintenance of MSCs on chitosan film via mTOR knockdown with mTOR specific shRNA. Upon lentiviral transduction with mTOR specific shRNA, MSCs decreased in the phosphorylation levels of mTOR, and its downstream molecules, such as S6, p-4E-BP1, and cleaved caspase 3 (Fig. 5a and Supplementary Figure S8a). The results showed that spheres formed by MSCs with mTOR knockdown decreased in size (Fig. 5b and Supplementary Figure S8b). Quantitative RT-PCR revealed that mTOR knockdown increased the mRNA levels of Oct4, Nanog and Sox2 (Fig. 5c). SA–β-Gal staining (Fig. 5d) also revealed the percentage of cells positive for SA–β-Gal activity was decreased in cells with mTOR knockdown (2.8 ± 0.8%) compared to cells without mTOR knockdown (4.9 ± 0.6%). Moreover, MSCs recovered from spheres formed by MSCs with mTOR knockdown, increased in osteogenesis when compared to MSCs recovered from spheres formed by MSCs transduced with control shRNAs (Fig. 5e). Similarly, we found knockdown of mTOR also reduced the dead cell numbers in the periphery of spheres (Fig. 5f). Together, these data suggest MSCs with mTOR knockdown decrease in sphere size, enhance in stem cell properties and reduce death in the periphery of spheres when cultured on chitosan film.

### Fibronectin was upregulated by mTOR and involved in sphere size regulation and dead cell attachment

**Figure 6:**
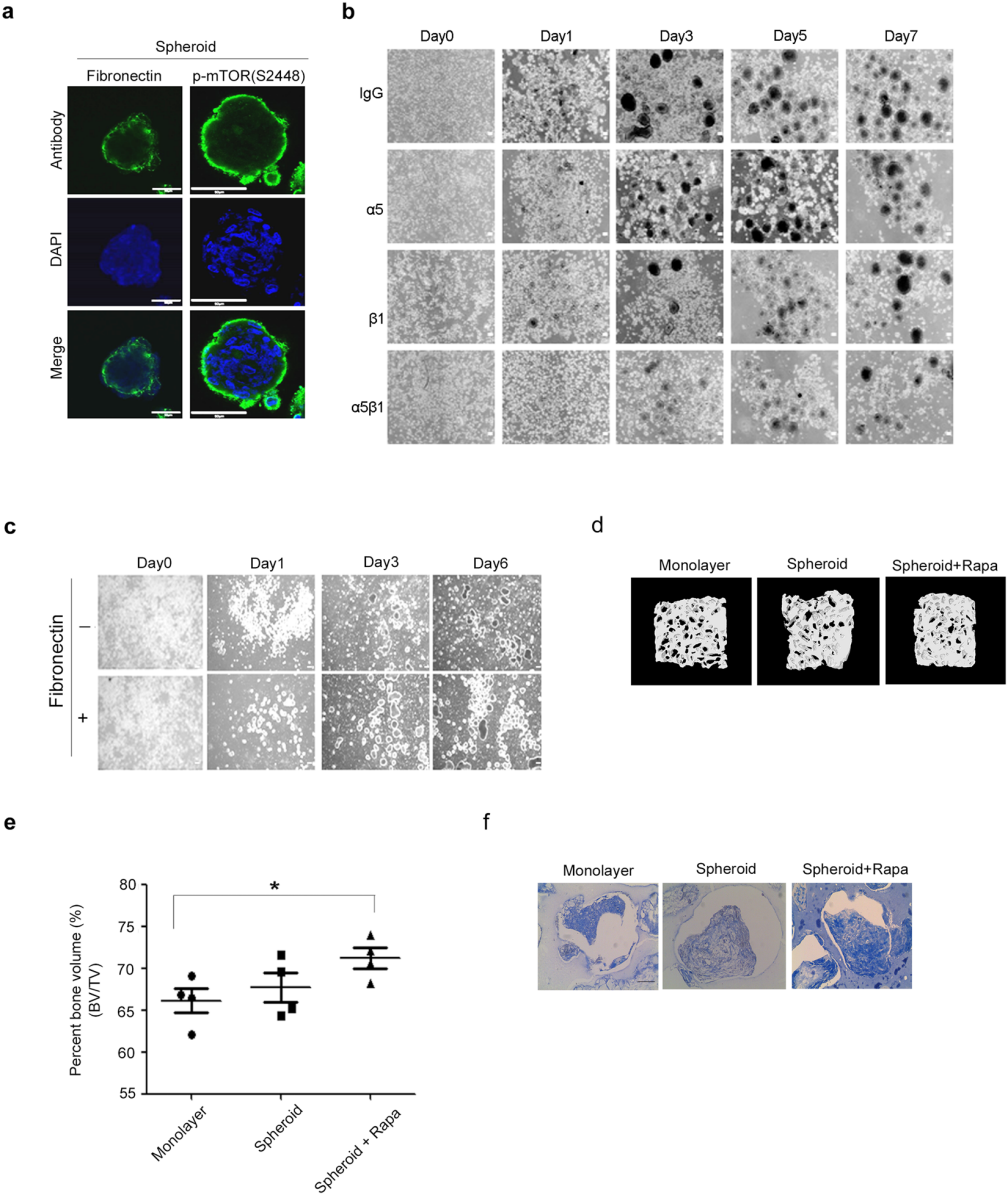
Sphere formation increased by chitosan film culture depends on fibronectin synthesis and interaction with α5β1 integrin. (**a**) Immunostaining for fibronection and phospho-mTOR expression in 3D sphere culture. All nuclei were stained with DAPI (Scale bar = 50 μM). (**b**) Representative pictures show the morphology of MSCs at indicated time points after chitosan film culture at the density of 2.5 × 10^4^/cm^2^ with neutralization antibodies against α5, β1 and α5β1. Isotype IgG was used as control (Scale bar = 100 μM). (**c**) Representative pictures show the morphology of MSCs at indicated time points after chitosan film culture with rapamycin at the density of 2.5 × 10^4^/cm^2^ in the absence or presence of recombinant fibronectin. Scale bar = 100 μM. Combination of chitosan film culture and rapamycin treatment increases the ability of MSCs to form bone *in vivo*. Evaluation of the percent bone volume and collagen deposition in an *in vivo* bone formation model. Aliquots of MSCs (1 × 10^6^) in monolayer culture, or in chitosan sphere culture without or with rapamycin treatment were delivered in ceramic cubes and induced in osteogenic induction medium for 1 week, followed by transplantation underneath the skin of immunodeficient mice (n = 4 for each group). At 8 weeks after implantation, transplants were subjected to micro-CT analysis (**d**) for the percent bone volume (**e**), and histological analysis by Mallory’s trichrome staining for collagen deposition (**f**). Scale bar = 100 μM. Results are presented as mean ± standard deviation. *p < 0.05 compared with monolayer cells. BV: bone volume; TV: total volume.

Because the mTOR-S6K-S6-4E-BP1 pathway has been known to increase protein synthesis^18^, we examined whether mTOR and its downstream signaling molecules increase sphere size via the production of certain extracellular matrix (ECM) and thereby induce dead cell attachment in the periphery. Because the mTOR-S6K-4E-BP1 pathway that is involved in protein synthesis was activated in the periphery of spheres, ECM synthesized by the spheres would be released into the conditioned medium of chitosan film culture. Through proteomics study of the conditioned medium, we found sphere culture on chitosan film increased in the secretion of several ECM proteins, with fibronectin as the most abundant compared to monolayer culture (Supplementary Figure 9a and Supplementary Table 1). Western blot analysis and immunofluorescence further revealed MSCs on chitosan film increased fibronectin expression in the periphery of the spheres (Supplementary Figure 9b and Fig. 6a). Moreover, inhibition of mTORC1 with rapamycin (Fig. 4a) or knockdown of mTOR (Fig. 5a) reduced the protein levels of fibronectin. Fibronectin-mediated cell attachment requires its interaction with integrin α5β1 on cell surface^19^. Interestingly, blocking integrin α5β1 interaction with neutralization antibodies against α5 or/and β1 reduced sphere size compared to isotype IgG (Fig. 6b). Moreover, addition of fibronectin in chitosan film culture treated with rapamycin increased sphere size (Fig. 6c). Together these data suggest the involvement of mTOR-S6K-S6-4E-BP1 in producing fibronectin for sphere size increase and the attachment of dead cells in the periphery of the spheres.

### Combination of chitosan film culture and rapamycin treatment for bone formation ***in vivo***

These data strongly suggest combination of chitosan film culture and rapamycin treatment selects more primitive MSCs in the spheres. These data urge us to ask whether these selective cells could be applied for new bone formation *in vivo*. To demonstrate this, MSCs harvested from monolayer culture or chitosan film culture in the absence or presence of rapamycin treatment were delivered in ceramic cubes, and induced for osteogenic differentiation for 1 week followed by transplantation into underneath the skin of immunodeficient mice for another eight weeks. Micro-CT analysis of transplanted constructs revealed that chitosan film sphere culture slightly increased the percentages of bone volume of the constructs compared to that of monolayer culture, while only combination of chitosan film sphere culture and rapamycin treatment significantly increased the percentages of bone volume of the constructs compared to that of monolayer culture (Fig. 6d and e). Histological evaluation of type I collagen synthesis with Masson’s Trichrome stain further revealed combination of chitosan film sphere culture and rapamycin treatment increased in type I collagen deposition compared to other groups (Fig. 6f). These data suggest combination of chitosan film culture and rapamycin treatment selects more primitive MSCs for increasing bone formation *in vivo*.

### Sphere culture activates mTORC1 to inactivate autophagy, thereby inducing cell death

**Figure 7:**
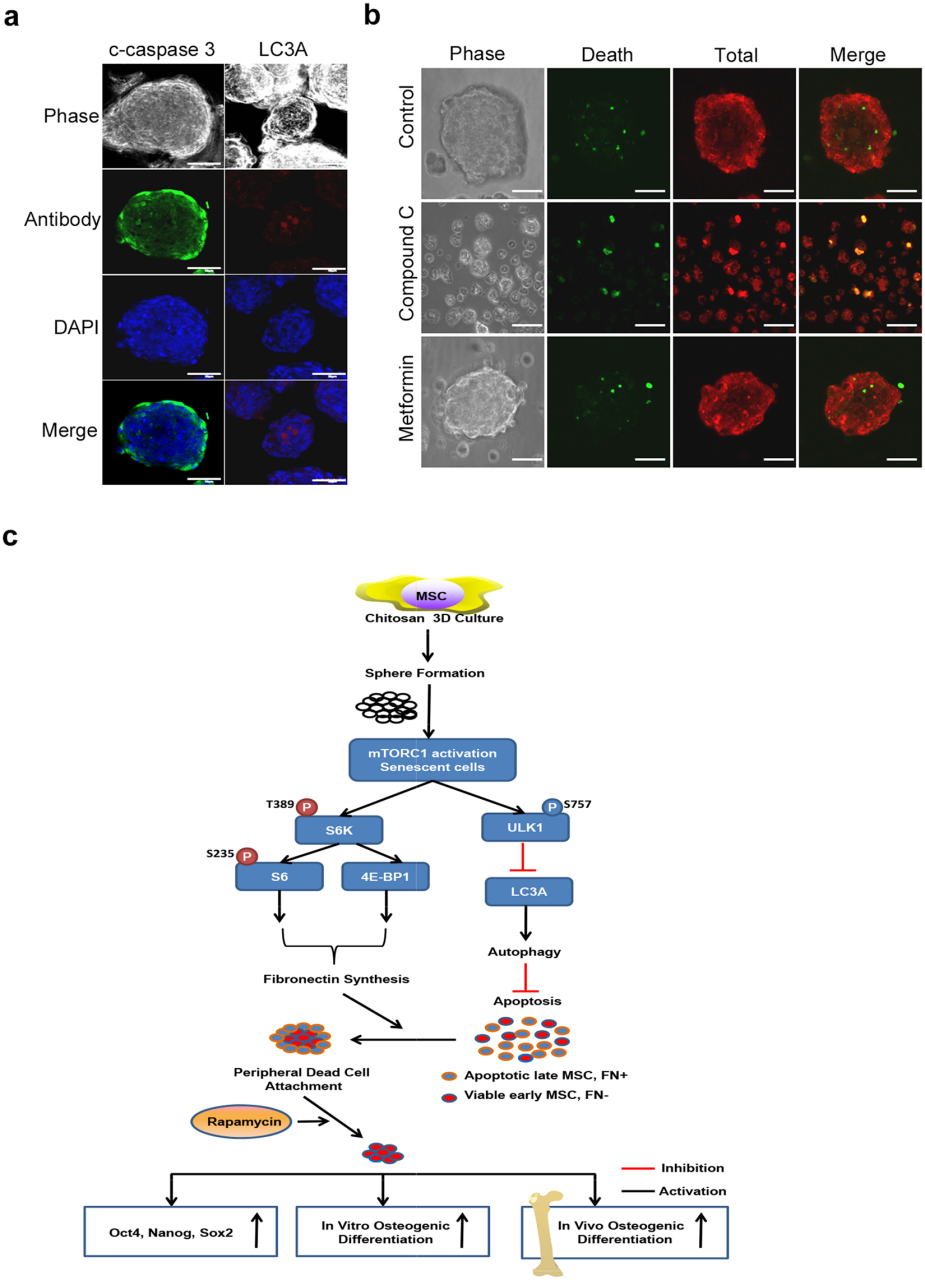
Chitosan film culture activates mTOR to activate S6K/S6/4EBP while inactivate Autophagy. (**a**) Cells were seeded at 2.5×10^4^/cm^2^ in dishes with chitosan coating for 48 hr, followed by immunostaining using antibodies against c-caspase 3 or LC3A. DAPI merged images are shown in the low panel (Scale bar = 50 μM). (**b**) The spheroid cells treated with compound c or metformin were then assayed for Live/Dead staining (Scale bar = 50 μM). (**c**) A scheme of the proposed model. Chitosan 3D sphere culture of MSCs enhances stem cell properties by selection of more primitive cells, which increase autophagy and thereby survive, while senescent cells decrease autophagy and undergo apoptosis. Especially in senescent cells, chitosan 3D sphere culture activates mTOR, which activates S6K/S6/4EBP1 to enhance fibronection synthesis and peripheral dead cell attachment, and phosphorylates ULK1 at S757 to further inactivate ULK1/Beclin and autophagy, thereby inducing apoptosis. Combination of chitosan 3D sphere culture with mTOR inhibition prevents peripheral dead cell attachment, thereby further increasing pluripotent gene expression, *in vitro* osteogenesis and *in vivo* bone formation.

To elucidate the underlying mechanism that chitosan film culture or mTORC1 pathway mediates to induce cell death, we chose to analyze the autophagy pathway including ULK1 and LC3A, which were known to be inactivated by mTORC1 while activated by rapamycin treatment and play a major role in inhibiting cell death in a lot of cells^20^. Western blotting analysis revealed that chitosan film culture increased the levels of S575 of ULK1 (inactive form) (Fig. 3b), which were reduced by rapamycin treatment (Fig. 4a) or mTOR knockdown (Fig. 5a). Immunofluorescence also showed LC3A punctate signals were located in the center of the spheres, while activated caspase 3 signaling were in the periphery (Fig. 7a). More importantly, inhibition of autophagy with compound C enhanced cell death, while stimulation of autophagy with Metformin, an AMPK activator^21^, inhibited cell death (Fig. 7b). Together these data suggest chitosan film culture activates mTOR to inactivate autophagy, thereby inducing cell death.

## Discussion

In the current study, we showed MSCs when cultured on chitosan film for 7 days formed 3D spheres, increased the expression of Oct4, Nanog and Sox2, and enhanced the osteogenic differentiation potential. We also found when cultured on chitosan film for 2 days, MSCs underwent significant apoptosis. Moreover, apoptosis was more obvious in late-passage MSCs compared to early-passage MSCs; this finding helps to explain why MSCs cultured on chitosan film increase pluripotent gene expression and enhance stem cell properties. Moreover, western blotting and immunostaining analyses revealed MSCs increased the activation of mTOR and its downstream molecules, S6K-S6 and 4E-BP1, which were involved in fibronectin synthesis and attachment of apoptotic cells in the periphery of the spheres. Combination of rapamycin treatment in chitosan film culture reduced fibronectin synthesis and inhibited the attachment of apoptotic cells in spheres, thereby reducing sphere size. Finally, we showed suppression of autophagy by chitosan film culture contributed cell death in chitosan film culture. The molecular mechanisms discovered here are novel and provide new insights into chitosan film culture of MSCs, which will help to develop new strategies in applying MSCs for therapeutics.

Previous data have shown that chitosan film culture increases sphere formation and stem cell properties in MSCs isolated from human adipose and placenta tissues^8, 9^. Chitosan film culture has been demonstrated to select multipotent MSC subpopulation from human gingival fibroblasts^22^. Chitosan or chitosan-hyaluronan film culture also promotes nanoparticle uptake and gene transfer efficiency for MSCs^23^. Most recently, Huang and Hus also demonstrate that chitosan-hyaluronan film culture increases epithelial-mesenchymal transition and cancer stem-like phenotypes in tumor spheres^24^. Besides the chitosan film, free chitosan in the medium also promotes the proliferation of osteoblasts and the osteogenic differentiation potential of MSCs^25^. These data suggest that chitosan, in the form of film, combined with other hydrogel, as well as the free form, is able to promote stem cell properties and therapeutic application of MSCs.

The exact mechanism that chitosan film mediated to induce spheroid formation or promote stem cell properties, however, remains mostly unknown, albeit several Wnt signaling pathways have been studied. For example, the calcium binding capacity of chitosan may affect the cell-substrate and cell-cell interactions and critically influence the dynamics of spheroid formation^26^. Chitosan film culture also upregulated the mRNA level of Wnt11, which was suggested to be involved in the spheroid formation and cardiomyogenic differentiation of MSCs on chitosan film. Wnt5A signaling and its crosstalk with other pathways such as ERK1/2 or Smad2/3 were shown to be involved in MSC assembly and sphere formation upon culturing in membranes made of chitosan or those further grafted with hyaluronan^27^. In the current study, we used a robust kinase protein assay to screen a panel of MAPK pathways and identified the mTOR signaling was the most upregulated pathway in chitosan film 3D sphere culture compared to 2D monolayer culture. Moreover, we used a rigorous method, including the use of chemical regulators and genetic modifiers, to demonstrate the involvement of mTORC1 and its downstream signaling pathways such as S6K-S6, 4E-BP1, ULK1, and LC3A in chitosan film culture-induced sphere formation, selection of more primitive stem cells by inducing cell death in senescent cells (Fig. 7C).

To be noted, cell apoptosis percentages of MSCs in chitosan film culture were different between Figs 1 and 2, which need to be discussed. MSCs at passage 3-4 were assayed in Fig. 1, while early primitive MSCs at passage 1–2 and late senescent MSCs at passage 6–7 were assayed in Fig. 2. The highest percentage of TUNEL positive cells was at 72 h of chitosan film culture for MSCs at passage 3–4, while the highest percentage of TUNEL positive cells was at 24 h of chitosan film culture for late senescent MSCs at passage 6–7. We speculate that the difference of the highest percentage of TUNEL positive cells between MSCs at passage 3–4 and that at passage 6–7 may be due to the different sensitivity of MSCs at different passage to suspension-induced apoptosis.

The effects of rapamycin and mTOR KD on affecting MSC stem cell properties and cell apoptosis or death in the current study seem similar, which needs further discussion. The mTOR kinase is present in two functionally and structurally distinct complexed termed TORC1 and TORC2, the former is rapamycin-sensitive, while the latter is not directly inhibited by rapamycin. Thus, rapamycin would mainly inhibited mTORC1 in the current study, while mTOR KD would suppressed both of mTORC1 and mTORC2. Because mTORC2 phosphorylates AKT to activate mTORC1 indirectly through inhibiting tuberous sclerosis complex (TSC)1/2, which negatively regulates mTORC1 signaling by converting Rheb (Ras homolog enriched in brain) into its inactive GDP-bound state^17^, therefore the end effect of mTORC2 activation by spheroid culture also leads to mTORC1 activation. In this study, evidence has shown that mTORC1 inactivation, either by rapamycin or mTORC1 knockdown, decreased sphere size, upregulated MSC stem cell properties and reduced dead cells in chitosan culture. However, we could not exclude the possibility that the effects of mTOR KD may work through decreasing mTORC2 activity, which needs to be clarified in the future. Interestingly, our data showed that LC3A punctate signals were located in the center of the spheres and the phosphorylated forms of mTOR, S6K, 4E-BP1 and activated caspase 3 signal were restricted to the periphery of the spheres. Because early primitive MSCs localizing in the sphere center, which were decreased in mTOR signaling, led to autophagy activation with increased LC3A staining, while late senescent cells attaching in the sphere periphery, which were increased in mTOR signaling, caused autophagy inactivation and subsequent cell apoptosis with activated caspase 3, thus it is anticipated that LC3A punctate signals were located in the center of the spheres and activated caspase 3 signal were in the periphery of the spheres. However, the reasons that early primitive MSCs localized in the sphere center, while late senescent cells attaching in the sphere peripheral should be clarified in the future.

There is debate as to whether chitosan film 3D sphere culture increases apoptosis or cell death compared to 2D monolayer culture. Previous studies found no significant apoptosis or cell death was observed in 3D sphere culture on chitosan film^8, 9^. However, in our study we used many methods, including live cell counting using the trypan blue exclusion method, TUNEL assay, Annexin V/PI flow cytometry, Live/Dead staining, western blotting analysis and immunofluorescence for cleavage of caspases, to demonstrate that chitosan film culture induces apoptosis in senescent cells, thereby selecting more primitive cells from the mixed MSC pool. These data were partly supported by some data of the previous studies where the Live/Dead staining at day 7 showed some dead cells in the periphery of the spheres^8^, and the video data showed some cell debris appeared immediately after the merge of different spheres^8, 9^. The discrepancy between this study and previous ones may be caused by the differences in observation time points and the inclusion of controls; the latter failed to check the early stages (day 2 and 3) of sphere culture and compare the results with 2D monolayer culture. Moreover, we identified the suppression of autophagy by mTORC1 as the pathway contributing to cell death in the periphery of the spheres. Through the suppression or knockdown of mTOR, we were able to select more primitive MSCs that are enriched in gene expression of pluripotency, *in vitro* osteogenesis and *in vivo* bone formation.

## Conclusion

These data successfully figure out the relationship between mTORC1 signaling and the chitosan film culture-mediated sphere formation, and increases in the expression of pluripotent genes, replicative and differentiation potential. Moreover, through this approach we develop new method to increase the stem cell properties and *in vivo* osteogenesis of MSCs, which will help develop novel strategies for therapeutic use of MSCs for bone tissue engineering.

## Materials and Methods

### Cell culture

The study was approved by the Institutional Review Board of Taipei Veterans General Hospital (#2012-05-007 A) and followed the guideline and regulation. The human primary MSCs were isolated from bone marrow aspirates of three individuals who signed the informed consents by a protocol described previously^28^. The cells were seeded at 100 cells per cm^2^ and grown in culture medium [(α-minimal essential medium; 1200-022, Gibco, Gaithersburg, MD), supplemented with 16.6% fetal bovine serum (FBS, Hyclone, Logan, UT), 100 units/mL penicillin, 100 μg/mL streptomycin (A5955, Sigma-Aldrich, St. Louis, MO), and 2 mM L-glutamine (G7513, Sigma-Aldrich), with medium change twice per week and a subculture was performed every 10 days. All of the experiments were performed with the MSC population isolated from the same donor, and repetitive experiments were performed using MSCs from different donors.

### Chitosan film preparation and culture

2.5 mL 1% (w/v) chitosan solution (C-3646, Sigma-Aldrich) dissolved in 0.67% (w/v) acetic acid (537020, Sigma-Aldrich) was added into each well of 6-well culture plates and air drying the plates in a hood for 24 hr to form a thin film, after which it was neutralized by 0.5 N NaOH aqueous solution (72064, Sigma-Aldrich) for 2 hr. Next, the plates were washed thoroughly with distilled water before being exposed to ultraviolet light overnight. The MSCs were seeded at the density of 2.4 × 10^5^ per well of 6-well plate. The cells were cultured at 37 °C in air with 5% CO_2_, and the medium was changed every 2 days.

### Cell counting for growth curves

All cells were seeded into 24-well culture plates (3 × 10^4^/well), the cells were allowed to continue in culture for 24 hr, 48 hr, or 72 hr and then were counted under the microscope with trypan blue (93595, Sigma-Aldrich) exclusion. All assays were performed in triplicate.

### Osteogenic differentiation

MSCs were induced in osteogenic induction medium [OIM: α-MEM supplemented with 16.6% FBS, 50 μg/mL ascorbate-2 phosphate (A8960, Sigma-Aldrich), 10^−8^ M dexamethasone (D1756, Sigma-Aldrich) and 10 mM β-glycerophosphate (50020, Fluka, Buchs, Switzerland)] for osteogenic differentiation. After the appearance of morphologic features of differentiation, cells were assayed for alizarin red S staining (A5533, Sigma-Aldrich), followed by optical density measurement of extracted dyes at 550 nm.

### Cell apoptosis assay

Detection of apoptotic cells was performed on cytospin preparations as well as on adherent cells cultured on chamber slides by using the TUNEL assay according to the manufacturer’s instructions (11-684-809-910, Roche, Mannheim, Germany). In brief, cells were harvested by trypsin treatment, stained with TUNEL assay, and visualized using Olympus fluorescence microscope (Tokyo, Japan).

### CFSE proliferation assay

Aliquots of 10^5^ MSCs were seeded in culture dishes and incubated with 1 mM CFSE solution (C43554, Invitrogen) for 15 min. The solution was then replaced with fresh medium. Detection of CFSE by flow cytometry (FACS Calibur, Becton Dickinson, San Jose, CA) was performed on the indicated days after culture.

### Annexin-V/PI flow cytometry

Flow cytometric analysis of Annexin-V and propidium iodide (PI) staining which detects the exposure of phosphatidylserine and the exposed DNA content in apoptotic cells, respectively was performed using an Annexin-V kit (1-858-777, Roche). All samples were incubated with PI and Annexin-V conjugated fluorescein isothiocyante (FITC) for 15-min then analyzed by FACS Calibur (Becton Dickinson), using CellQuest (Becton Dickinson) and FlowJo software (TreeStar, Ashland, OR) for data analysis.

### Live/Dead staining

For analysis cell proliferation/survival, all cells were plated at 1 × 10^5^ cells per well into multi-well plate. The Live/Dead double staining kit was used according to the supplier’s recommendation (Enzo Life Sciences, Farmingdale, NY). The kit uses a cell-permeable green fluorescent to stain dead cells and an impermeable red fluorescent dye to stain all cells.

### SA–β-gal staining

Cells were washed with PBS and fixed with 2% formaldehyde/0.2% glutaraldehyde. After washing, the cells were incubated at 37 °C for an appropriate time with fresh β-Gal chromogenic substrate solution (1 mg/mL; X-Gal; V394A, Cell Signaling), 40 mM citric acid [pH 6.0], 5 mM potassium ferrocyanide, 5 mM potassium ferricyanide, 150 mM NaCl, and 2 mM MgCl_2_). All reagents were purchased from Sigma-Aldrich. The experiment was repeated three times and the number of cells expressing β-gal was calculated.

### Protein extraction and western blotting analysis

Cell extracts were prepared with M-PER (78501, Pierce, Rockford, IL) plus protease inhibitor cocktail (78442, Pierce) and protein concentrations were determined using the BCA assay (23225, Pierce). Protein lysates (30 μg) were separated on SDS polyacrylamide gels, transferred onto PVDF membranes, blocked with 5% blotting grade milk (1706404, Bio-Rad, Hercules, CA) in TBST (20 mM Tris-HCl [pH 7.6], 137 mM NaCl, 1% Tween 20). The membranes were incubated with the primary antibodies overnight at 4 °C. After extensive washing, the membranes were further incubated with horseradish peroxidase-conjugated secondary antibodies for 1 hr. Immunoreactive proteins were then developed using an enhanced chemiluminescence detection system (PerkinElmer, Boston, MA). Membranes were exposed to X-ray film (Amersham Pharmacia Biotech, Piscataway, NJ) for visualization. Primary antibodies include anti-phospho-mTOR (Ser2448) (1:1000, BS4706, Bioworld, St Louis, MN), anti-cleaved caspase-3 (1:1000, E83-77, Epitomics), anti-fibronectin (1:5000, SC-8422, Santa Cruz, San Diego, CA), anti-phospho-ULK1 (ser757) (1:1000, 6888, Cell Signaling, Danvers, MA), anti-phospho-S6K (Thr389) (1:1000, 9234, Cell Signaling), anti-cleaved caspase-9 (1:1000, 9502, Cell Signaling), anti-phospho-S6 (Ser235/236) (1:1000, 2211, Cell Signaling), anti-LC3A (1:1000, 4599, Cell Signaling), and phospho-4E-BP1 (1:1000, 2855, Cell Signaling). An anti-β actin antibody (1:5000, NB600-501, Novus, Littleton, CO) was used as loading control.

### Immunofluorescence

For immunofluorescence staining, specimens were fixed in PBS with 4% formaldehyde, washed twice with PBS, and immersed in 0.1% Triton X-100 overnight at 4 °C, followed by washing 3 times with PBS. The following primary antibodies were incubated overnight at 4 °C: anti-phospho-mTOR (Ser2448) (1:200, Bioworld), anti-cleaved caspase-3 (1:200, Epitomics), anti-fibronectin (1:200, Santa Cruz) anti-phospho-ULK1 (ser757) (1:1000, Cell Signaling), anti-phospho-S6 (Ser235/236) (1:200, Cell Signaling) or anti-LC3A (1:50, Cell Signaling). After incubation with primary antibodies, cells were washed with PBS and then incubated with fluorescence-conjugated secondary antibodies (1:5000, FITC, Jackson ImmunoResearch, Westgrove, PA) for 2 hr at room temperature. After nuclear staining with 40,6-diamidino-2-phenylindole (DAPI, D9542, Sigma-Aldrich), spheres were then placed in a Lab-Tek^®^II chamber (Nunc, Naperville, IL) and analyzed with a confocal fluorescent microscope (Olympus FV10i). Negative controls without utilizing primary antibodies were also prepared to rule out nonspecific labeling.

### Lentiviral vector production and cell infection

All the RNAi reagents were obtained from the National RNAi Core Facility supported by the National Science Council in Taiwan. Expression plasmids and the bacteria clone for mTOR shRNA (Accession number: NM 004958; TRCN0000038677 and TRCN0000199321) were used to generate recombinant lentiviral particles. Sub-confluent cells were infected with lentivirus. At 24 hr post-infection, we remove medium and replaced with fresh growth medium containing puromycin (3 μg/mL; P8883, Sigma-Aldrich) to select infected cells for 48 hr.

### RNA extraction and quantitative real-time polymerase chain reaction

Total RNA was isolated with TRIzol reagent (15596, Invitrogen) and cDNA was synthesized from 2 μg of total RNA with oligo-dT using Superscript III cDNA synthesis kit (18080-093, Invitrogen) for 30 min at 50 °C, followed by 2 min at 94 °C to inactivate the reverse transcriptase. The expression levels of *Oct4*, *Nanog*, *Sox2* and *GAPDH* in all sample were assessed by real-time PCR using SYBR Green (04913914001, Roche) with following cycling conditions: 95 °C for 10 min, 40 cycles of 95 °C for 15 sec, 60 °C for 1 min, and 72 °C for 20 sec. Standard curves (cycle threshold values versus template concentration) were prepared for each target gene and for the endogenous reference [glyceraldehyde 3-phosphate dehydrogenase (GAPDH)] in each sample. Quantification of unknown samples was performed using LightCycler Relative Quantification Software version 3.3. The following primers sequences were used: *GAPDH* (NM_001289745.1) Forward primer 5′-CTCTGCTCCTCCTGTTGTTCGACA-3′; Reverse primer 5′-ACGACCAAATCCGTTGACTC-3′; *Sox2* (NM_003106) Forward primer 5′-ATGCACCGCTACGACGTCA-3′; Reverse primer 5′-CTT TTGCACCCCTCCCAT TTT-3′; *Nanog* (XM_011520852.1) Forward primer 5′-ATGCCTGTGTTTGTGGGCC-3′; Reverse primer 5′-GCCAGTTGTTTTTCTGCCAC-3′; *Oct4* (NM_001159542.1) Forward primer 5′-AGCCCTCATTTCACCAGGCC-3′; Reverse primer 5′-CAA AACCCGGAGGAGTCCCA-3′.

### Membrane microarray assay

Human phospho-MAPK antibody array membranes (ARY002B, R&D, Minneapolis, MN) consisting of 26 unique capture MAPKs and other serine/threonine kinases antibodies in duplicate were used according to the manufacture’s instruction. The detailed protocol and sensitivity thresholds can be found in the manufacturer’s website (R&D). The intensity of cytokines was indicated as relative intensity following the formula: Sample was minus to negative control.

### ***In vivo*** osteogenic differentiation

All experimental protocols involving mice were approved by the Institutional Animal Care and Use Committee of Taipei Veteran General Hospital and performed in accordance with the relevant guidelines and regulations. Female nude mice, 8–10 weeks old were purchased from BioLasco Taiwan Co., Ltd. Aliquots of 10^6^ cells were delivered in 5 × 5 × 5 mm ceramic cubes (ZE10.10, Zimmer, Warsaw, IN) followed by induction with OIM. One week after induction, the cell-containing cubes were transplanted subcutaneously into immunodeficient mice for another 8 weeks. The specimens were harvested for percent bone volume (BV) and total volume (TV) analysis by SkyScan 1076 (micro-CT, Bruker, Brussels, Belgium) and collagen deposition assessment by Mallory trichrome staining (AABFB001, American MasterTech, Lodi, CA).

### Statistical analysis

GraphPad Prism 5 software was used for statistical analysis. All values were expressed as mean ± standard deviation. Comparisons between two groups were analyzed by Student’s t-test, otherwise (more than three groups) were analyzed by one way ANOVA. A value of P < 0.05 was considered statistically significant.

## Electronic supplementary material

Supplementary Figures and Tables
