# Supplementary material for: Involvement of mTOR-autophagy in the selection of primitive mesenchymal stem cells in chitosan film 3-dimensional culture
Source: Sci Rep. 2017 Aug 31;7:10113. doi: 10.1038/s41598-017-10708-0 (PMC5578982; doi:10.1038/s41598-017-10708-0)
Supplement: Supplementary file 1 — Supplementary Figures and Tables [file 41598_2017_10708_MOESM1_ESM.doc]

**Involvement of mTOR-autophagy in the selection of primitive mesenchymal stem cells in chitosan film 3-dimensional culture**

Hsiao-Ying Chiu Ph.D, Yeou-Guang Tsay M.D., Ph.D., Shih-Chieh Hung M.D., Ph.D.

**Inventory of supplementary information**

**Supplementary Figures**

Supplementary Figures S1

Supplementary Figures S2

Supplementary Figures S3

Supplementary Figures S4

Supplementary Figures S5

Supplementary Figures S6

Supplementary Figures S7

Supplementary Figures S8

Supplementary Figures S9

**Supplementary Table**

Supplementary Table S1

**
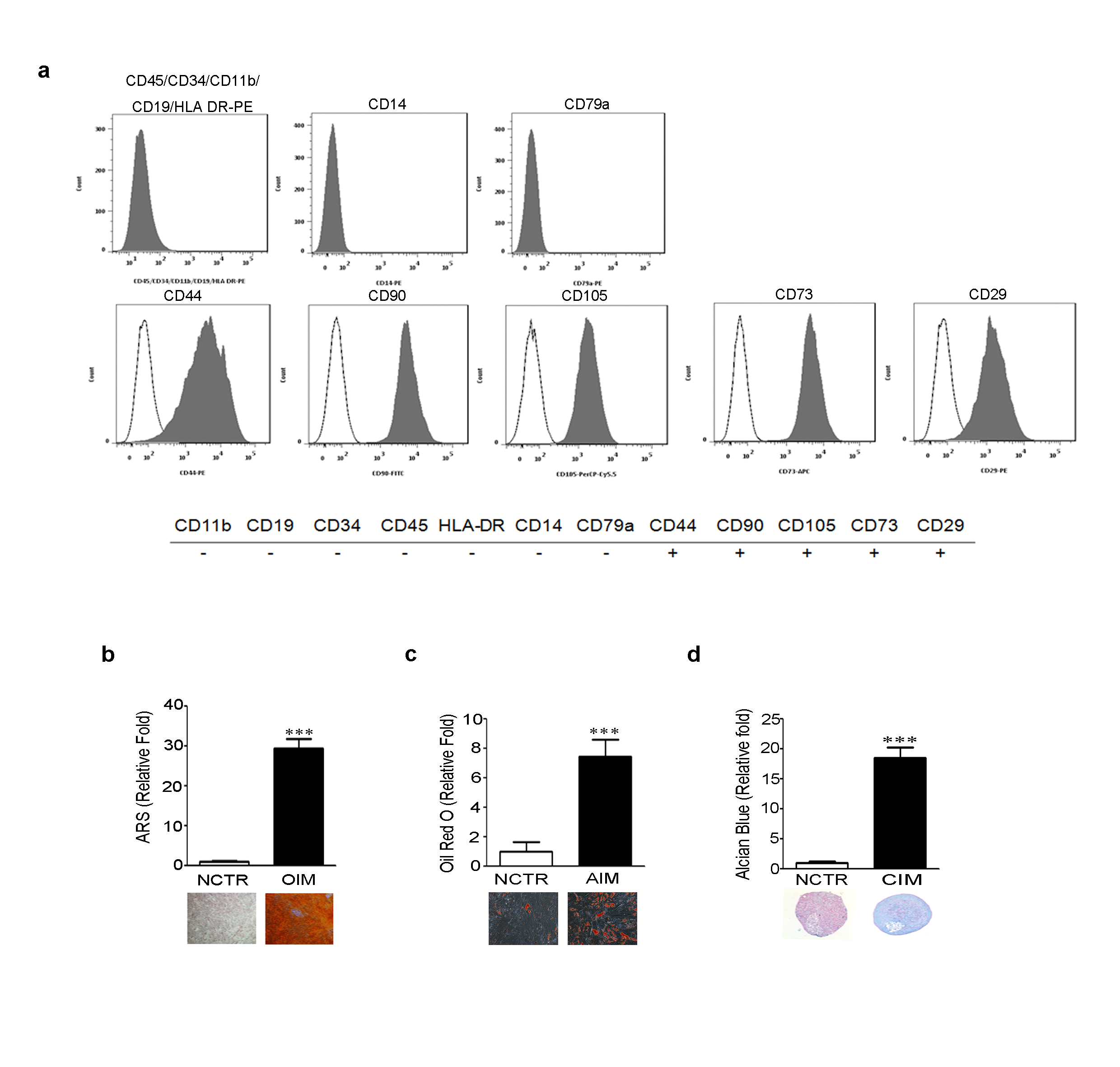
**

**Supplementary Figure S1. Characterization of MSCs.** MSCs were expanded at low density culture (100 cells/cm^2^) and subculture was performed every 10 days. Representative data of MSCs at passage 3 show (**a**) the surface marker profile and (**b-d**) the multi-differentiation potentials into (**b**) osteoblasts, (**c**) adipocytes, and (**d**) chondrocytes. The results are expressed as the mean ± standard deviation of three independent experiments. Asterisks indicate significant differences. AIM: adipogenic induction medium; OIM: osteogenic induction medium; CIM: chondrogenic induction medium.

**
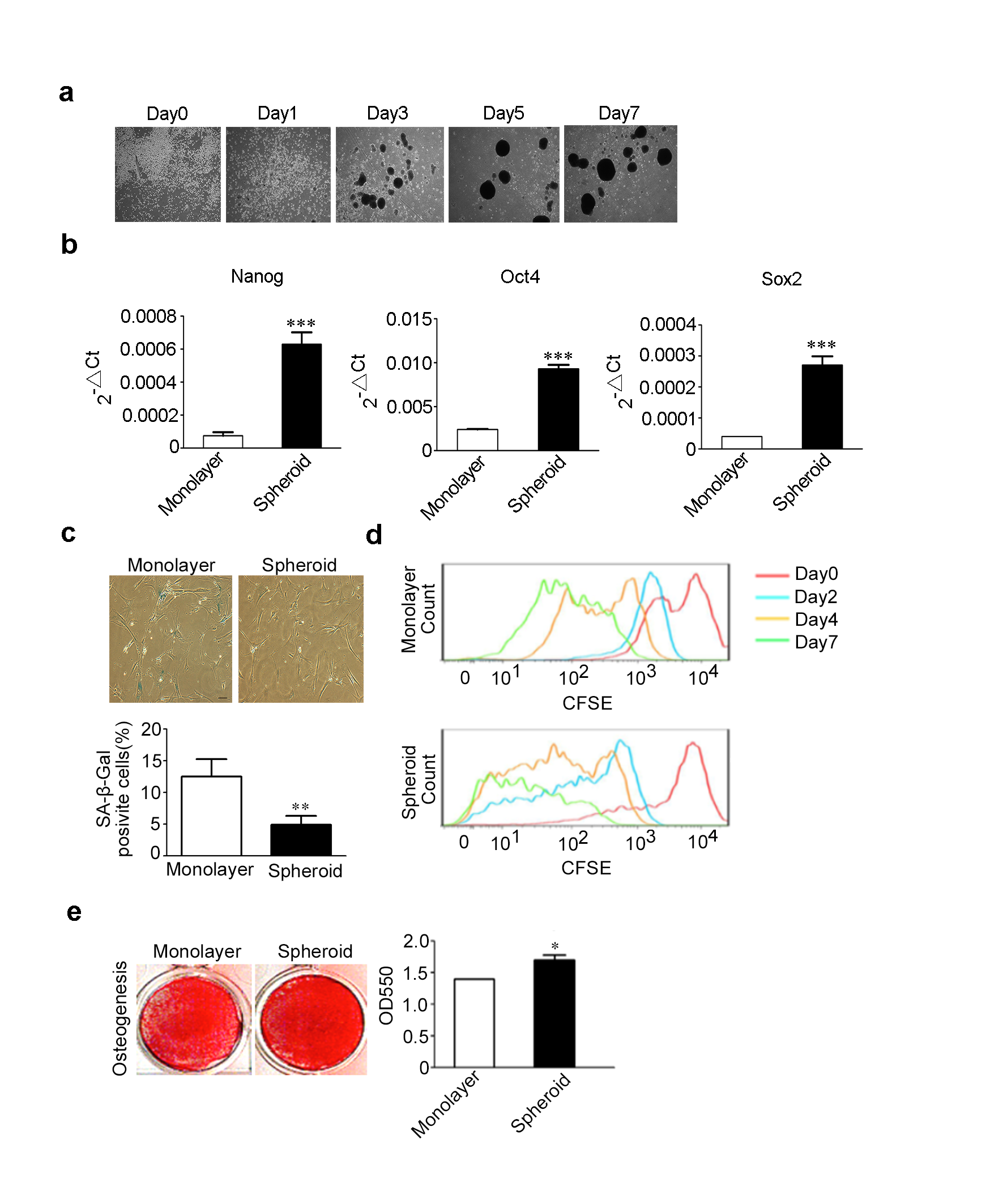
**

**Supplementary Figure S2. Chitosan film culture increases sphere formation and enhances stem cell properties in MSCs.** (**a**) MSCs (passage 3~4) were seeded in dishes coated with chitosan at 2.5×10^4^ cells/cm^2^ for indicated time points. The representative morphologies are shown in the pictures. Scale bar =200μM. (**b**) Quantitative RT-PCR reveals higher expression of pluripotent genes in MSCs cultured on chitosan film (Spheroid) compared to MSCs cultured without chitosan coating (Monolayer). The △Ct was normalized to GAPDH. (**c**) Chitosan film culture decreases in senescence marker expression. MSCs recovered from 7-day chitosan film culture and monolayer culture were reseeded for 2 days, followed by measurement of senescence-associated β-galactosidase (SA-β-Gal) staining. Scale bar =100μM. Upper panel: representative images of cells stained with SA-β-Gal. Lower panel: Percentage of cells positive for SA-β-Gal staining. (**d**) MSCs were labeled with Carboxyfluorescein Diacetate Succinimidyl Ester (CFSE) to monitor cell proliferation before seeding in chitosan film culture and monolayer culture for indicated time points, followed by flow cytometric analysis of fluorescence retention. (**e**) After 7 days of seeding, cells were reseeded and induced for osteogenic differentiation for 14 days, followed by alizarin red S staining (Left panel) and optical density measurement of extracted dyes at 550nm (Right panel). The results are expressed as the mean ± standard deviation of three independent experiments. Asterisks indicate significant differences. *p<0.05, **p<0.01 and ***p<0.005 compared with Monolayer cells.

**
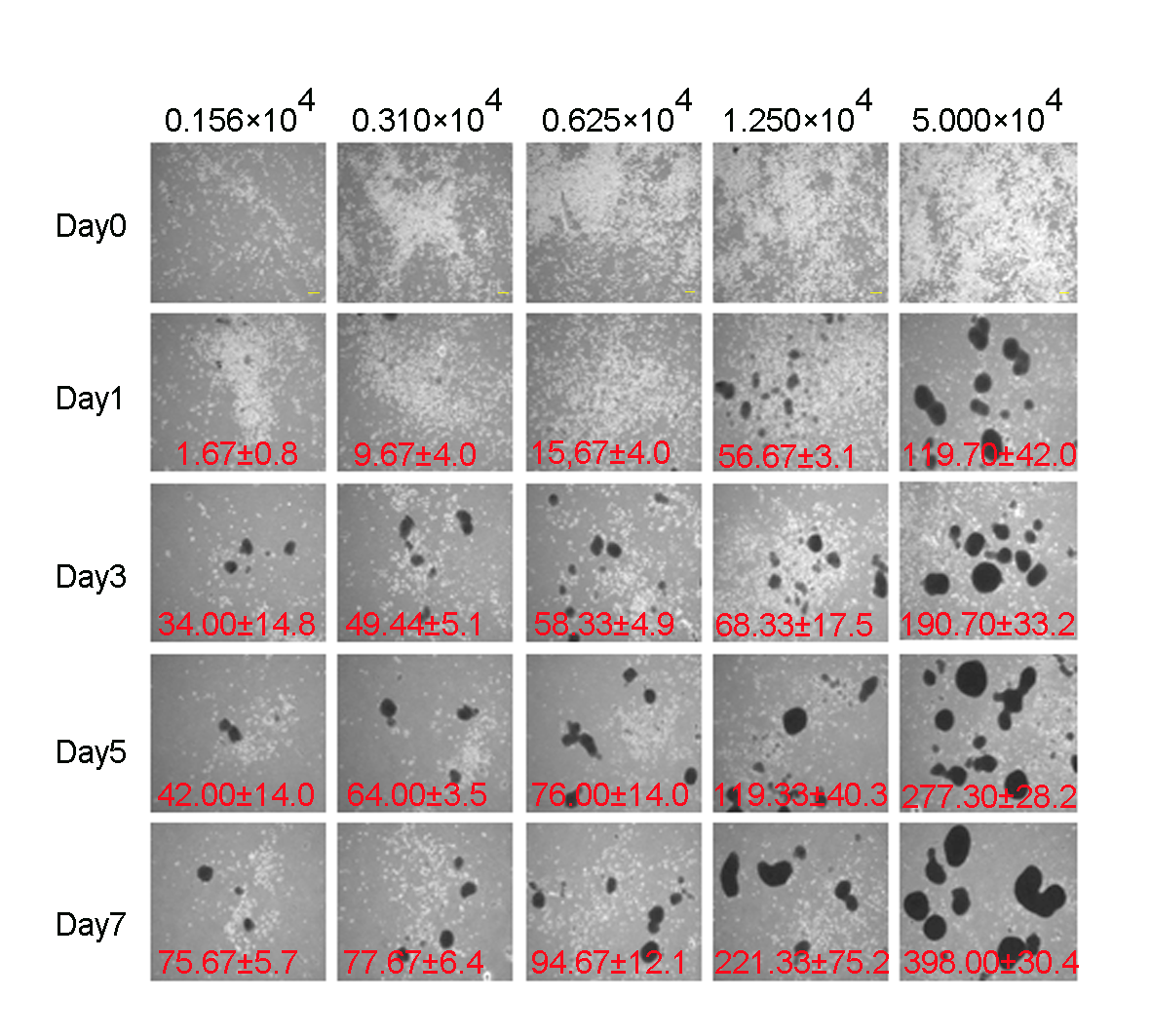
**

**Supplementary Figure S3: The size of spheres correlated with the cell density.** MSCs (passage 3~4) were seeded in petri dishes coated with chitosan at indicated density for indicated time points. The representative morphologies are shown in the pictures. Sphere size was measured (mean ± standard deviation). Scale bar =200 μM.


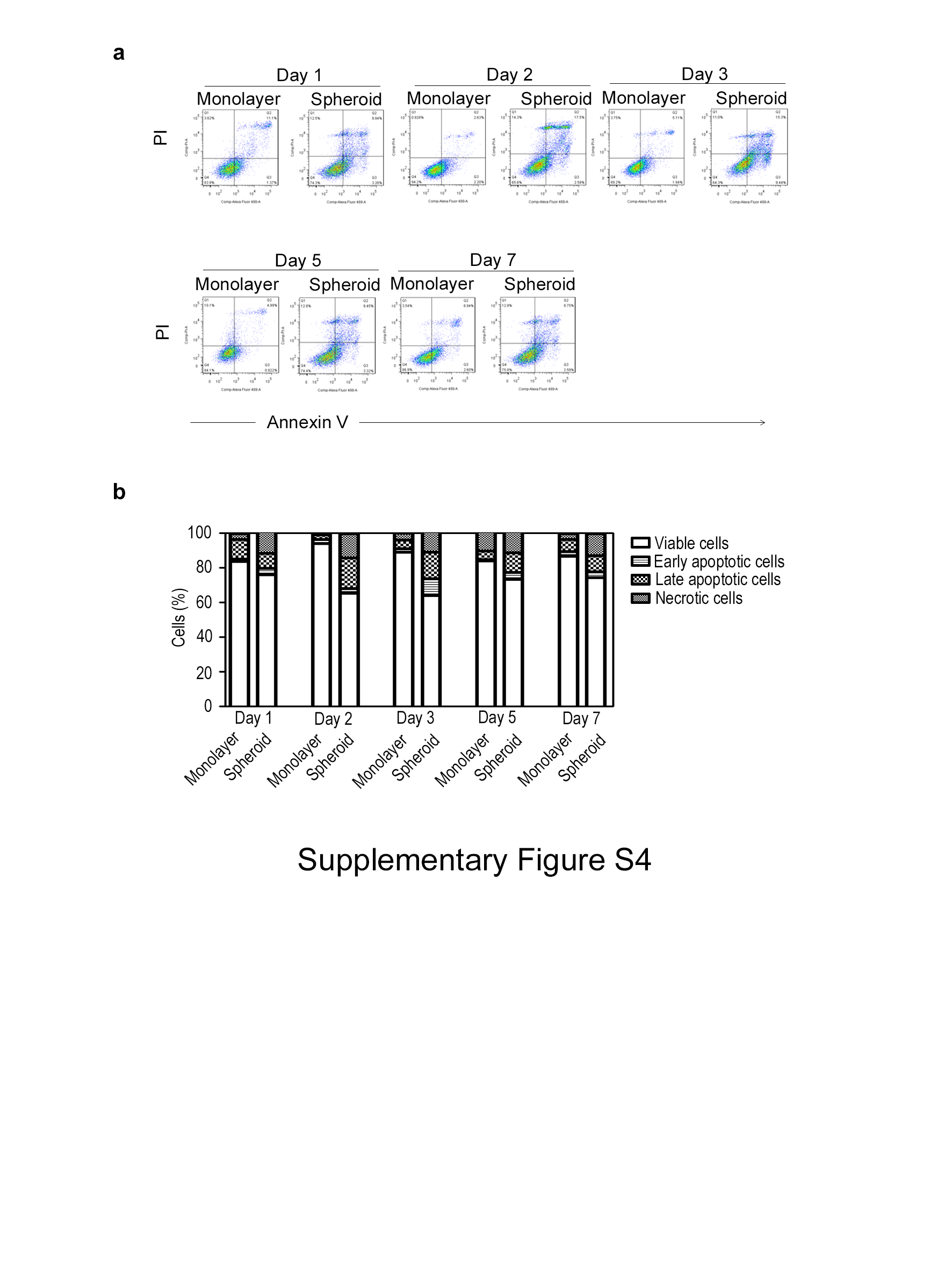


**Supplementary Figure S4. The spheroid formation on chitosan film is associated with an increase in apoptosis.** MSCs (passage 3~4) were seeded at 2.5×10^4^/cm^2^ without (Monolayer) or with chitosan coating (Spheroid), followed by Annexin V/PI assay at indicated time points. (**a**) the representative of Annexin V/PI assay; (**b**) the quantitative data.


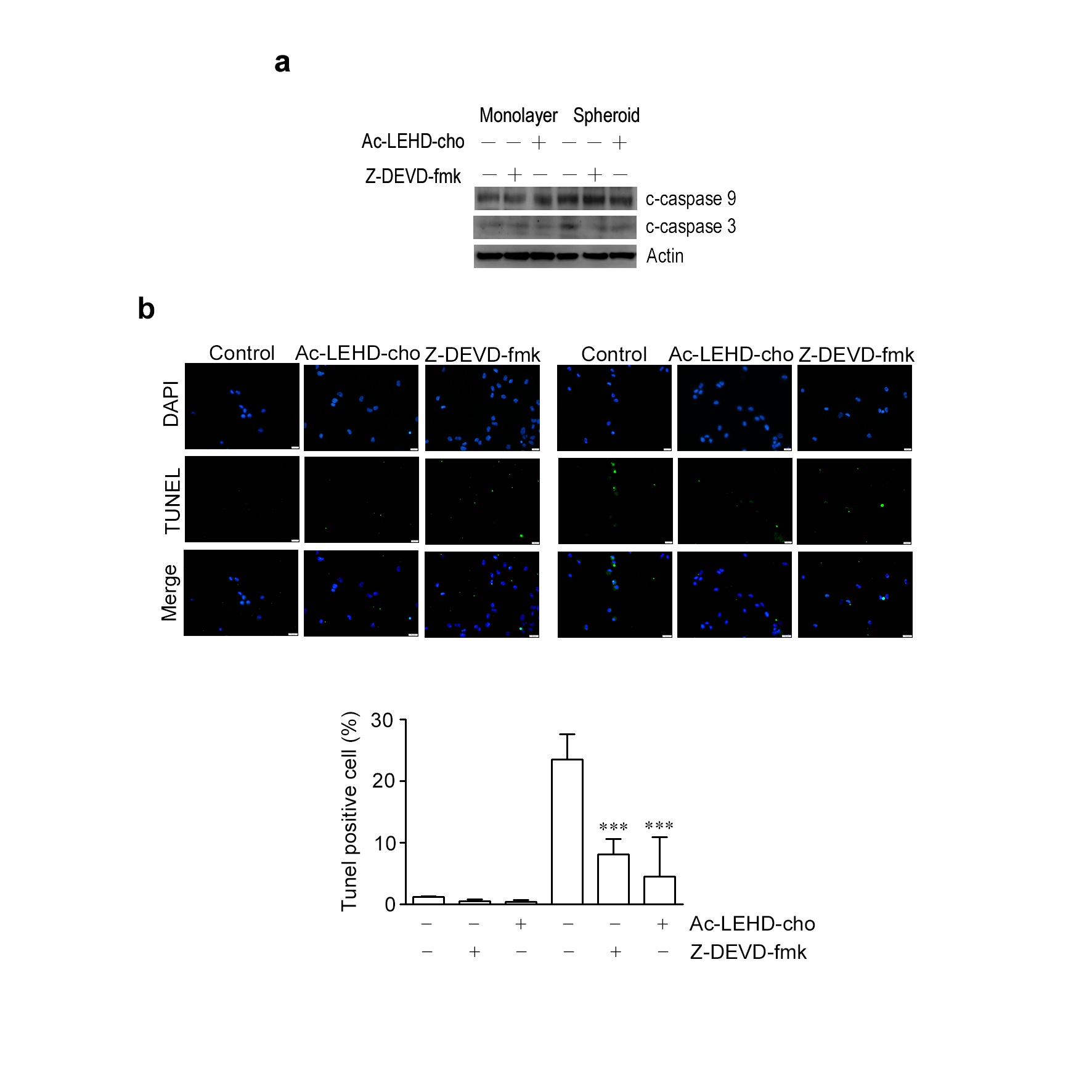


**Supplementary Figure S5. Caspase inhibitors block chitosan film culture-induced apoptosis.** MSCs at passage 3 were pretreated without or with caspase 3 inhibitor, Z-DEVD-fmk (20μM), or caspase 9 inhibitor, Ac-LEHD-cho (20μM) for 1 hr, followed by seeding to chitosan film culture for 48 hr. (**a**) Whole-cell lysates at 48 hr were analyzed by western blotting with specific antibodies against c-caspase-9 and c-caspase 3. Actin was used as a loading control. (**b**) Cells were subjected to TUNEL staining (Upper panel) and the percentage of positive cells was counted (Lower panel). The results are expressed as the mean ± standard deviation of three independent experiments. ***p<0.005 compared with cells without pretreatment of caspase inhibitors as determined with student’s t test.


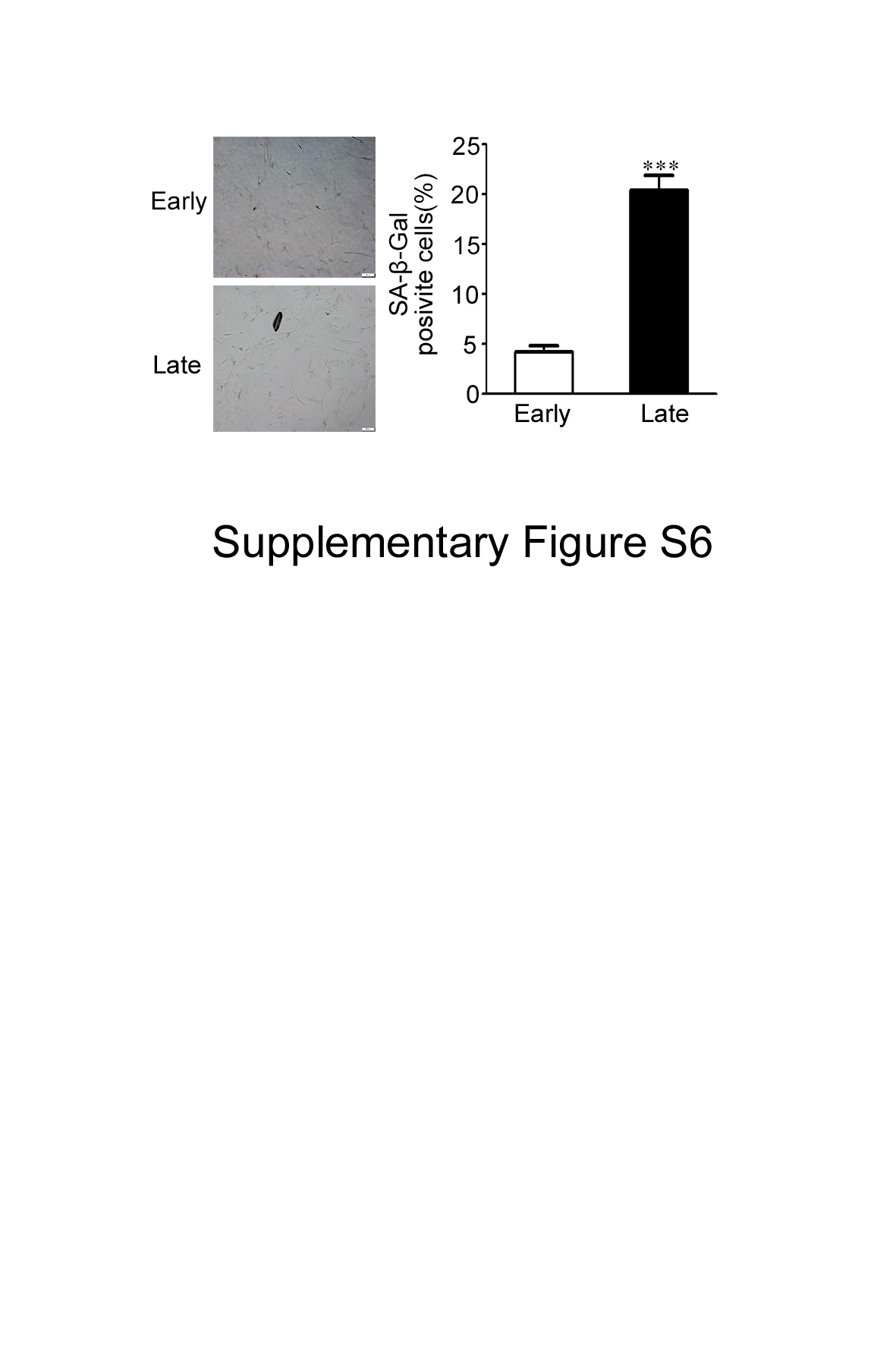


**Supplementary Figure S6. Late passage MSCs increase in SA-β-Gal activity.** MSCs of early (passage 1-2) and late passages (passage 6-7) were subjected to SA-β-Gal staining (Left panel) followed by counting of positive percentage (Right panel). The results are expressed as the mean ± standard deviation of three independent experiments. ***p<0.005 compared with early passage as determined with student’s t test.


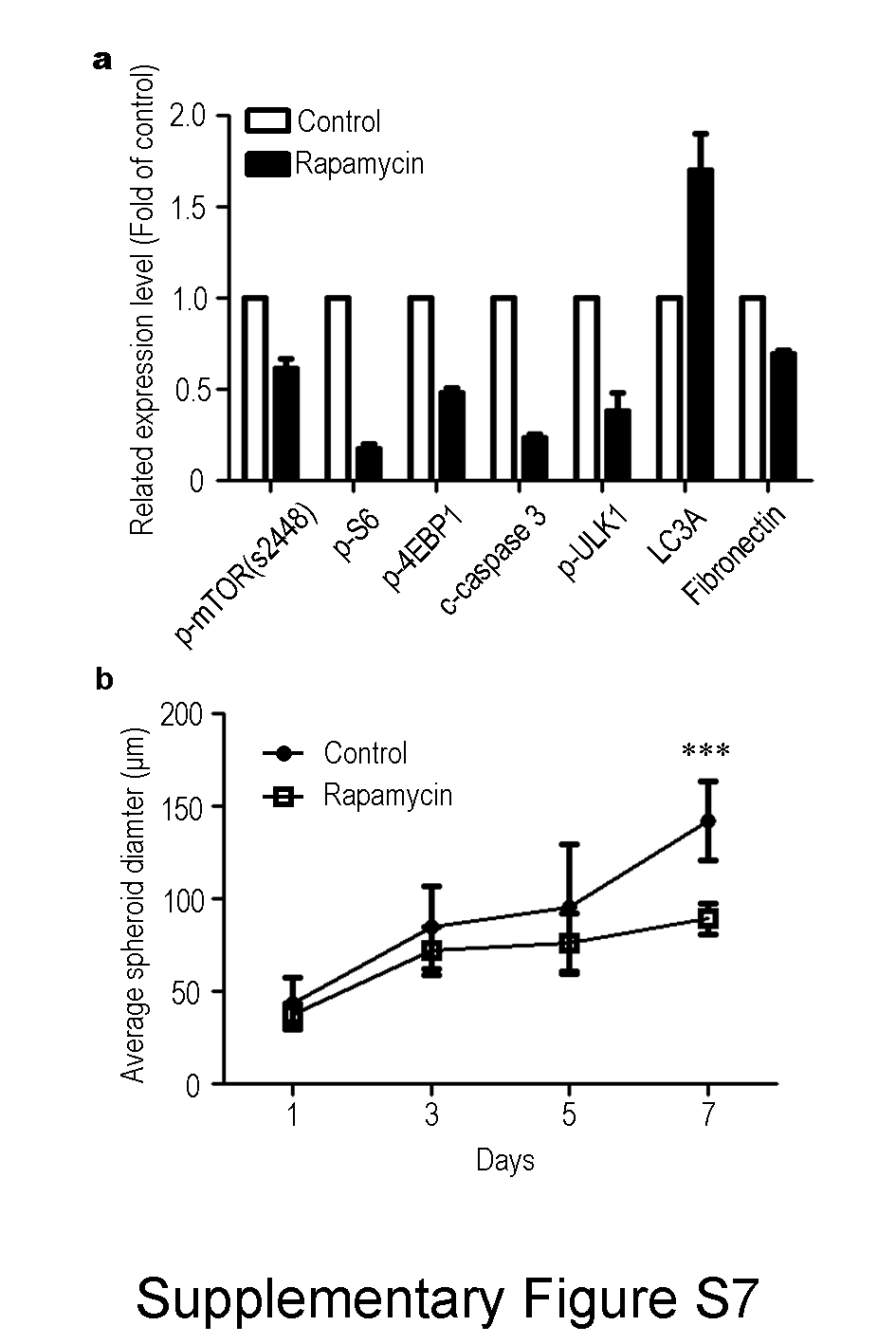


**Supplementary Figure S7. Rapamycin treatment in chitosan film culture decreases sphere formation and downstream molecules protein.** MSCs (passage 3~4) were seeded at 2.5×10^4^/cm^2^ in dishes coated with chitosan in the absence or presence of rapamycin. (**a**) After 48 hr of seeding on monolayer or with chitosan coating (Spheroid), cell lysates were analyzed by western blotting and quantification of all related signaling proteins. β-tubulin was used as a loading control. The relative ratio to control (in the absence of rapamycin) is shown. The results (mean ± standard deviation) are quantitative data of cell lysates from three individuals. (**b**) Representative sphere size at indicated time points. The results are expressed as the mean ± standard deviation of three independent experiments, which is representative of MSCs from two individuals. ***p<0.005 compared with rapamycin treatment as determined with two way ANOVA.

**
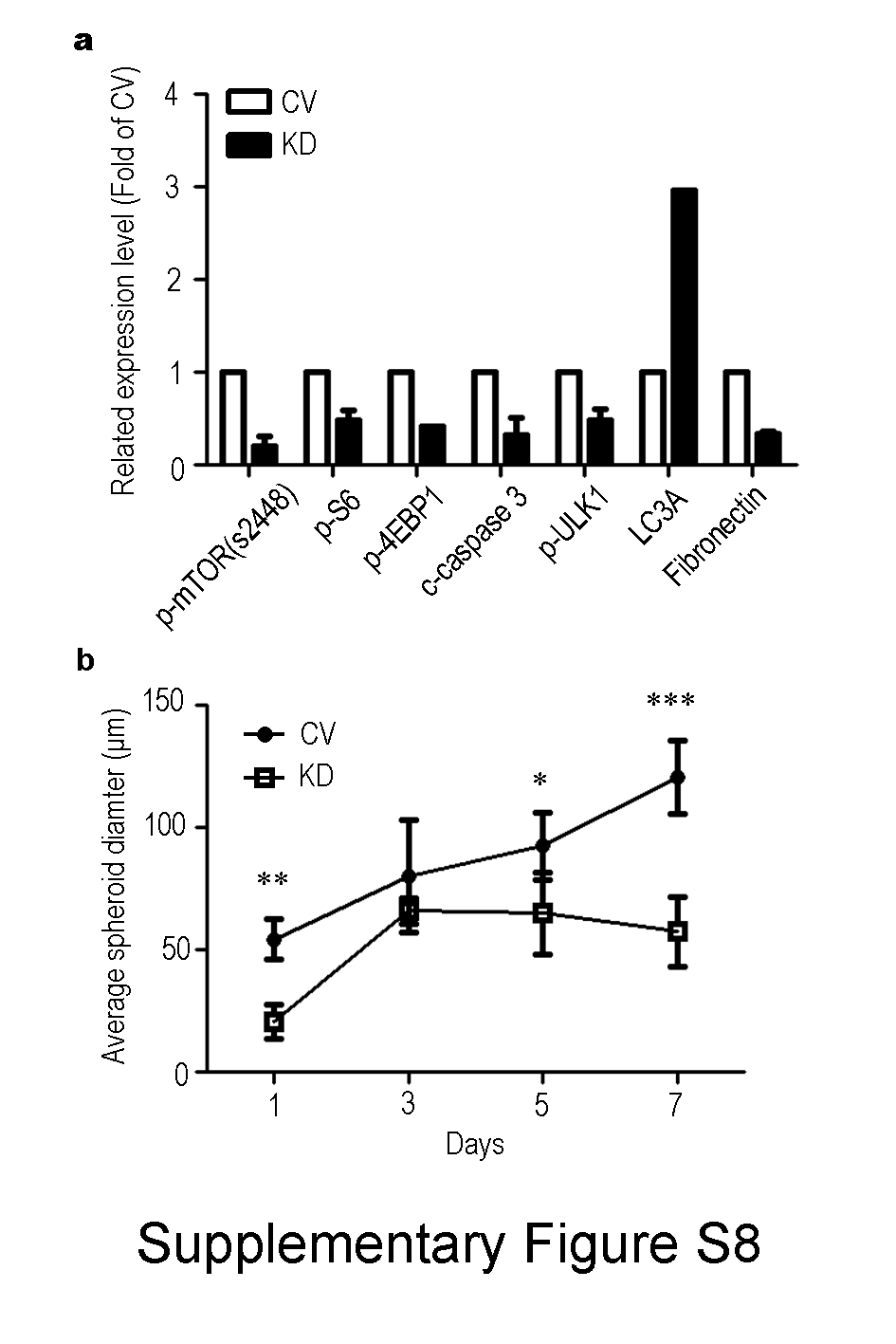
**

**Supplementary Figure S8. Knockdown of mTOR in chitosan film culture decreases sphere formation and downstream molecules protein.** (**a**) MSCs (passage 3~4) were transfected with shRNAs against mTOR (mTOR KD) and seeded in dishes coated with chitosan at 2.5×10^4^/cm^2^. Transfection with non-specific shRNAs (control vector, CV) was used as a control. After 48 hr of seeding, cell lysates were assayed using western blotting analysis and quantification of all related signaling proteins. β-tubulin was used as a loading control. The relative ratio to control vector is shown. The results (mean ± standard deviation) are quantitative data of cell lysates from three individuals. (**b**) Representative sphere size at indicated time points. The results are expressed as the mean ± standard deviation of three independent experiments, which is representative of MSCs from two individuals. *p<0.05, **p<0.01, ***p<0.005 compared with mTOR KD as determined with two way ANOVA.


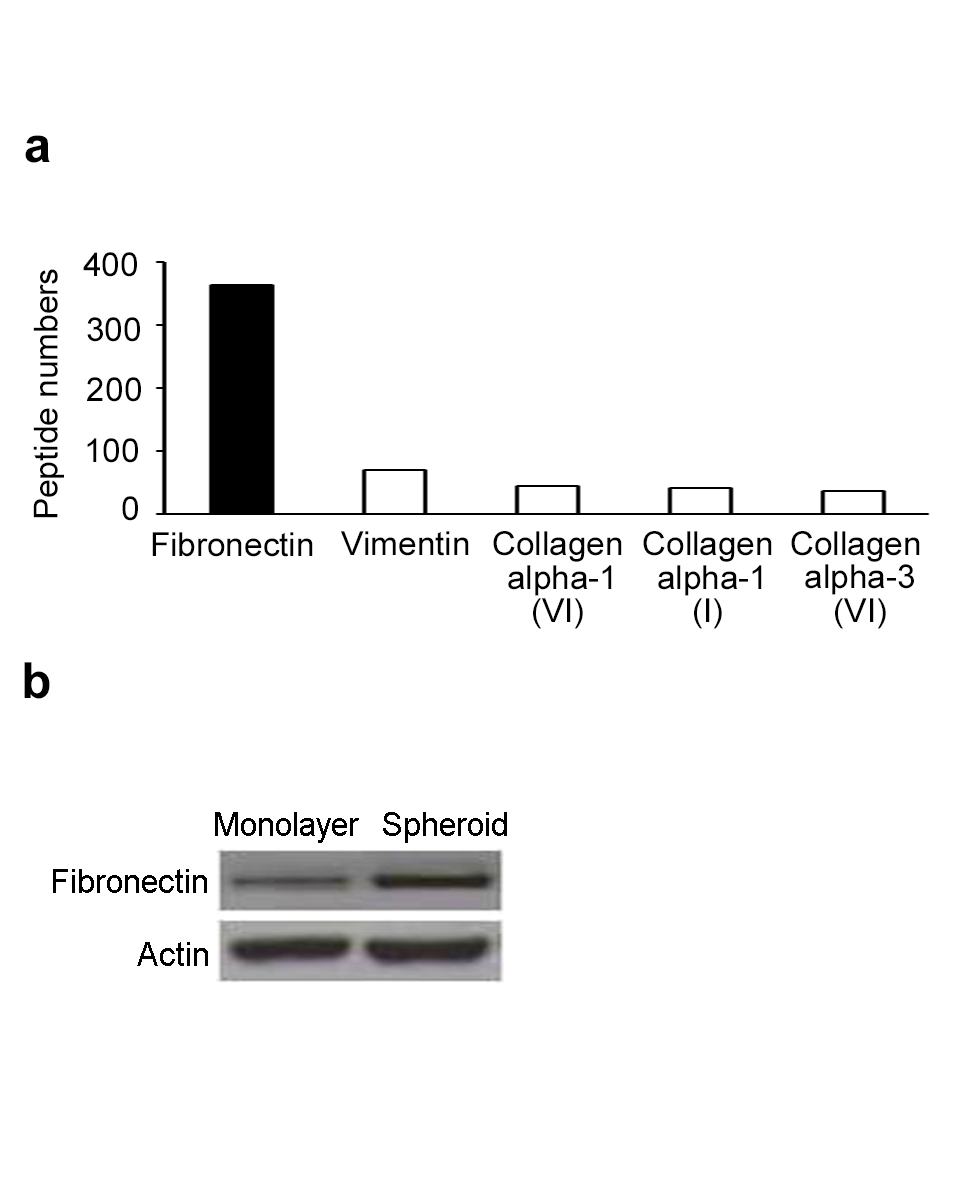


**Supplementary Figure S9. Sphere culture on chitosan film increased in the secretion with fibronectin as the most abundant compared to monolayer culture.** **(a)** MSCs (passage 3~4) were seeded at 2.5×10^4^/cm^2^ in dishes with chitosan coating for 48 hr (Spheroid), followed by the replacement of medium with serum-free basal medium. After 72 hr of medium replacement, aliquots of conditioned medium were collected and assayed for proteomic analysis. Protein clustering analysis revealed 247 proteins were enriched in the conditioned medium of 3D sphere culture. Among them, the most five enriched ECM molecules are shown here. (**b**) Whole-cell lysates were used for western blot analysis using specific antibodies against fibronectin. Actin was used as a loading control.

**
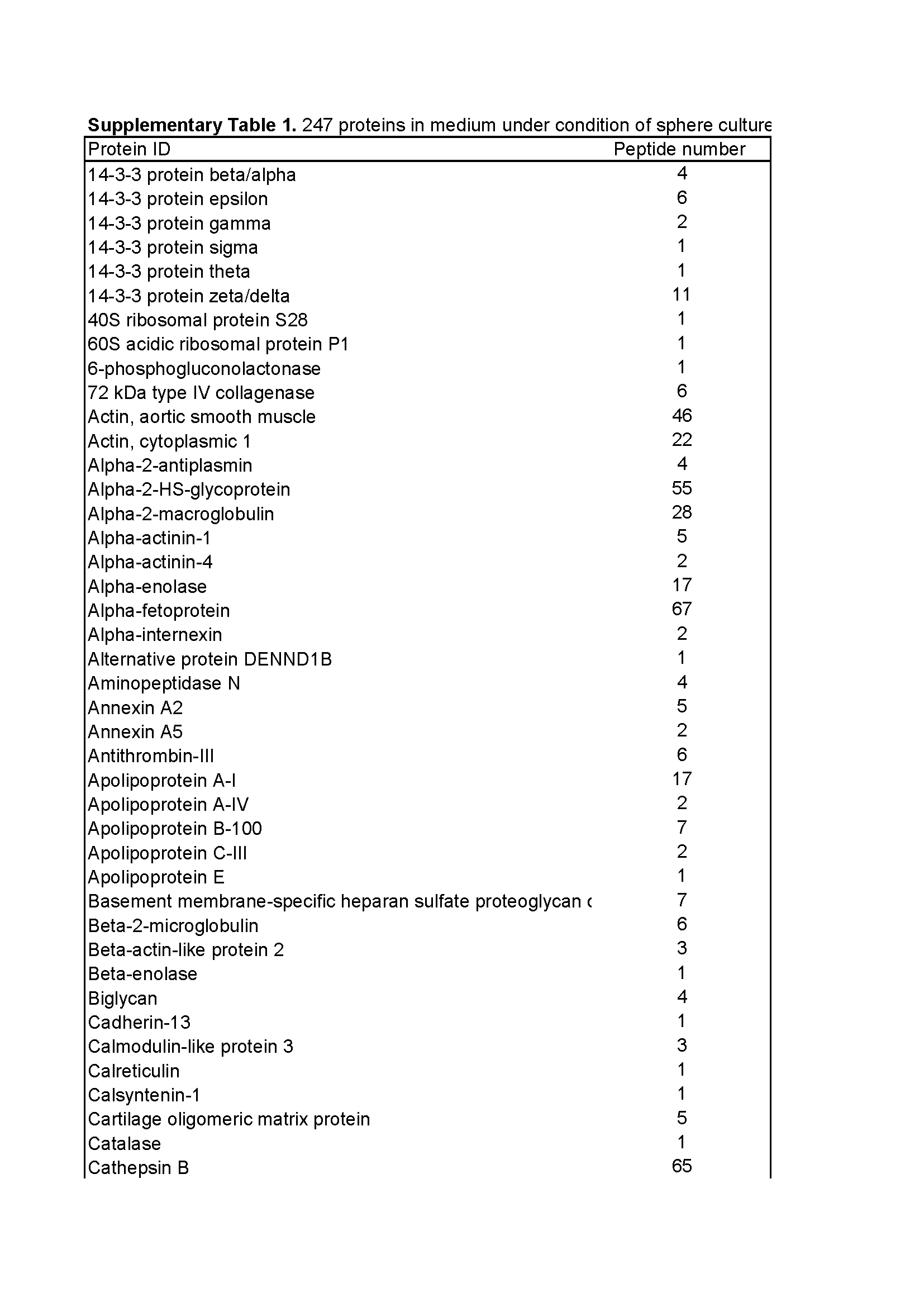

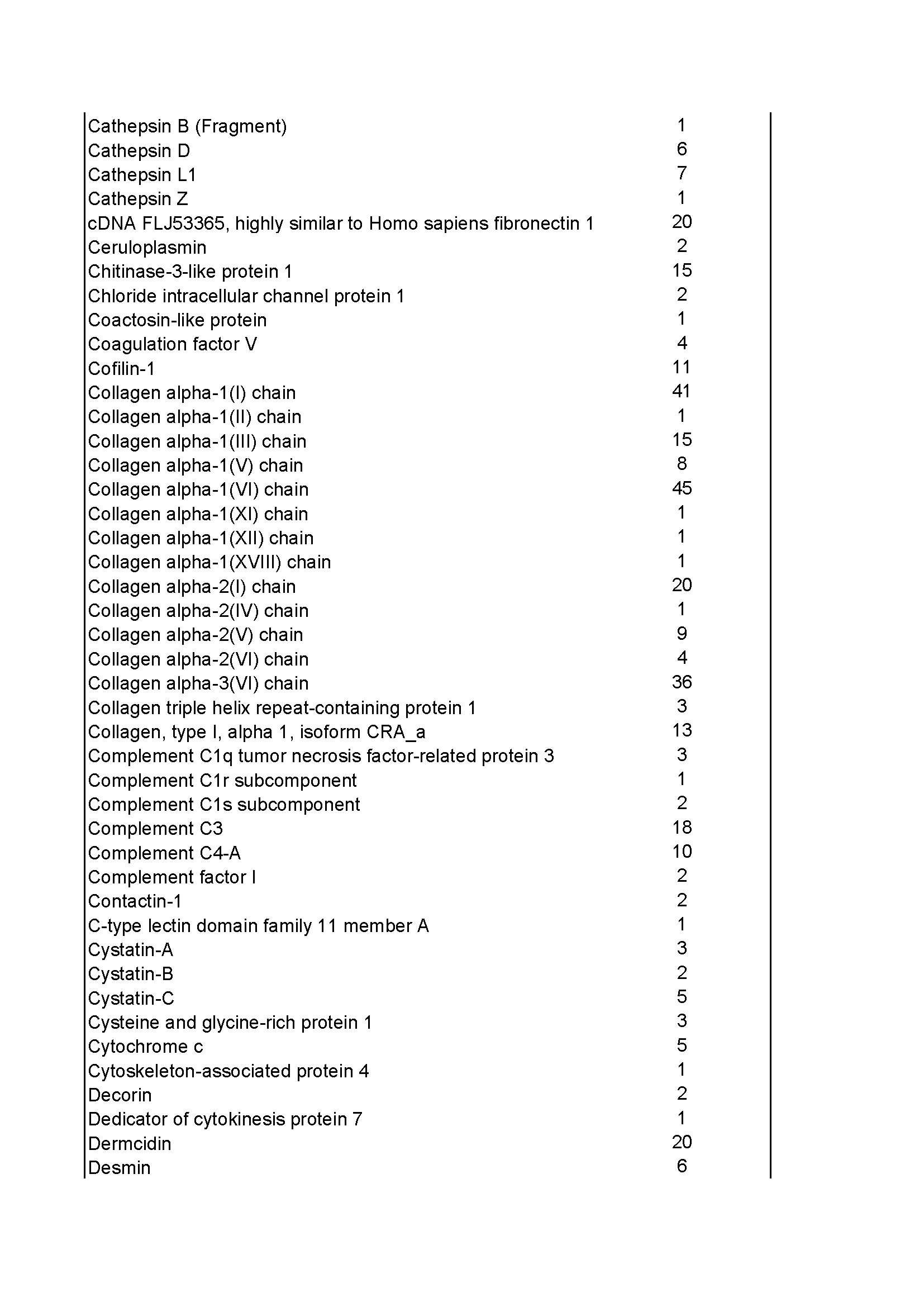

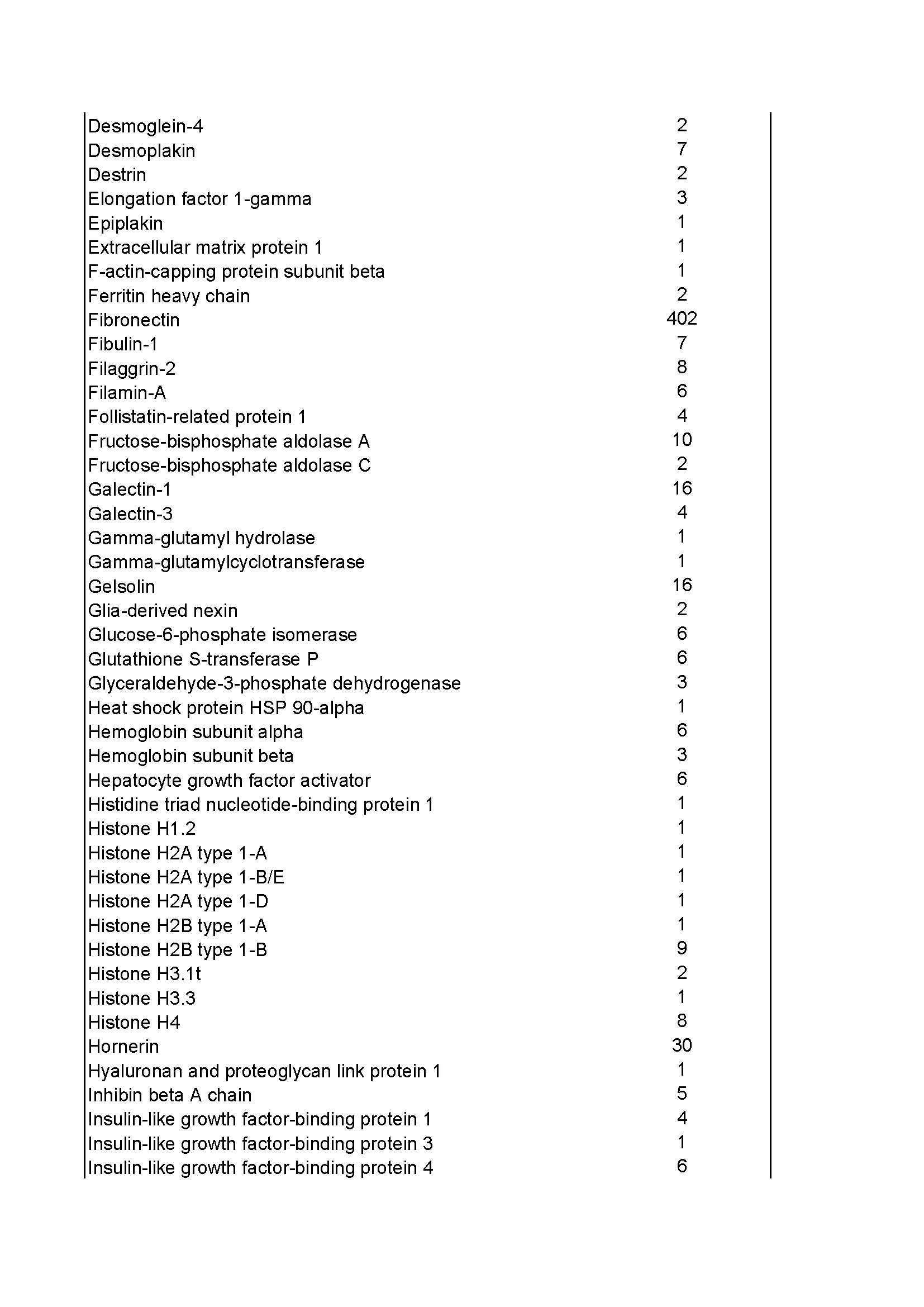

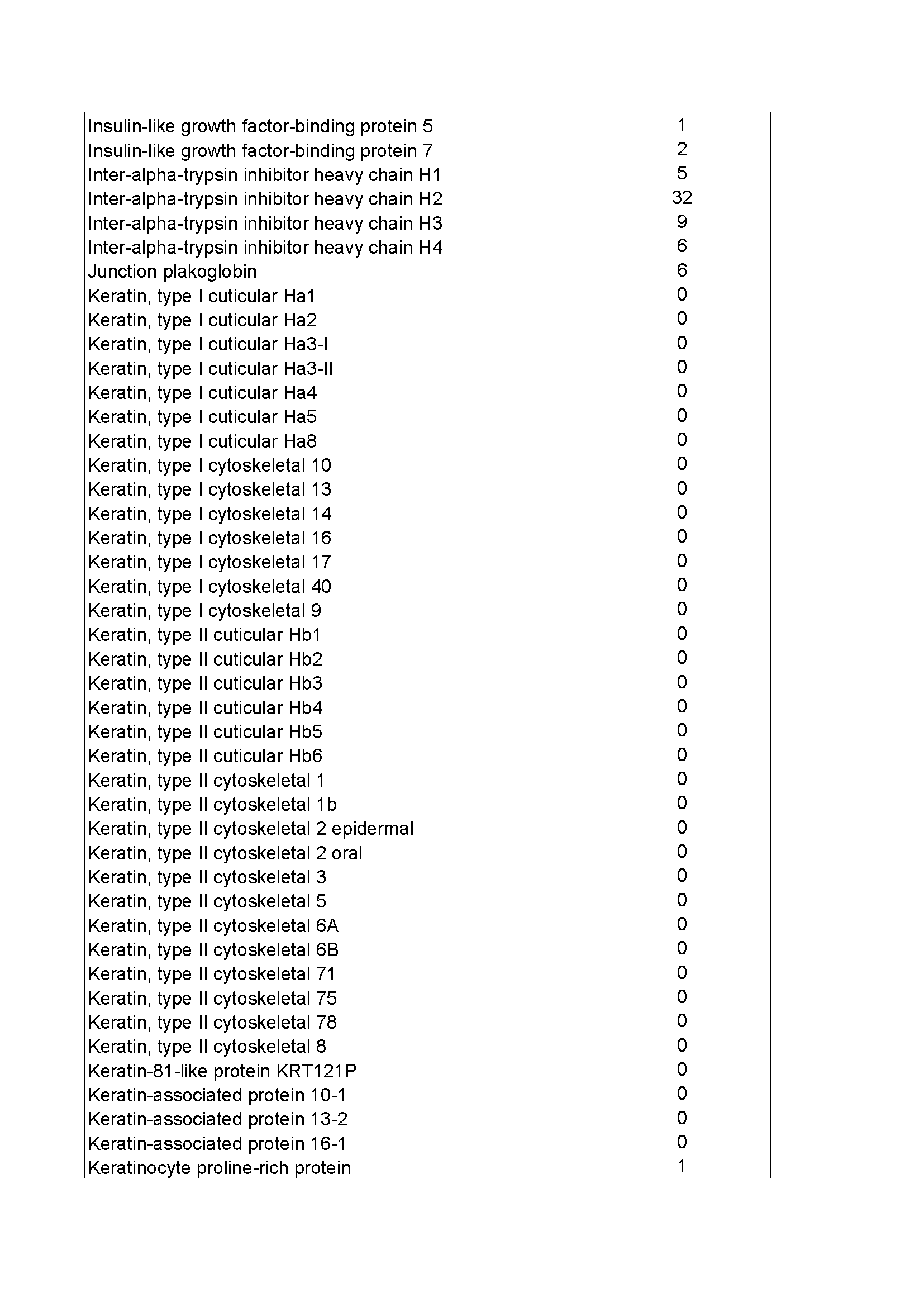

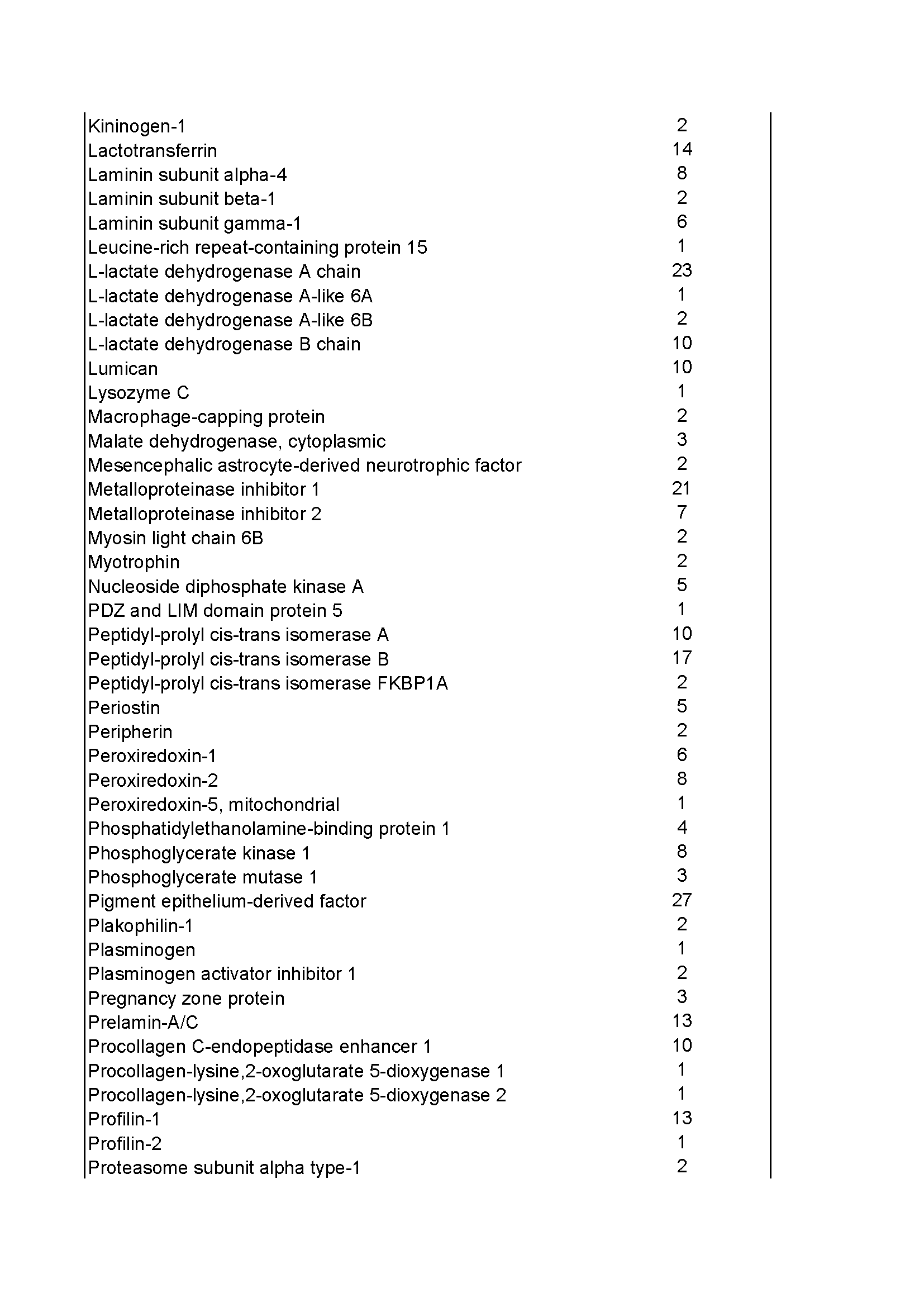

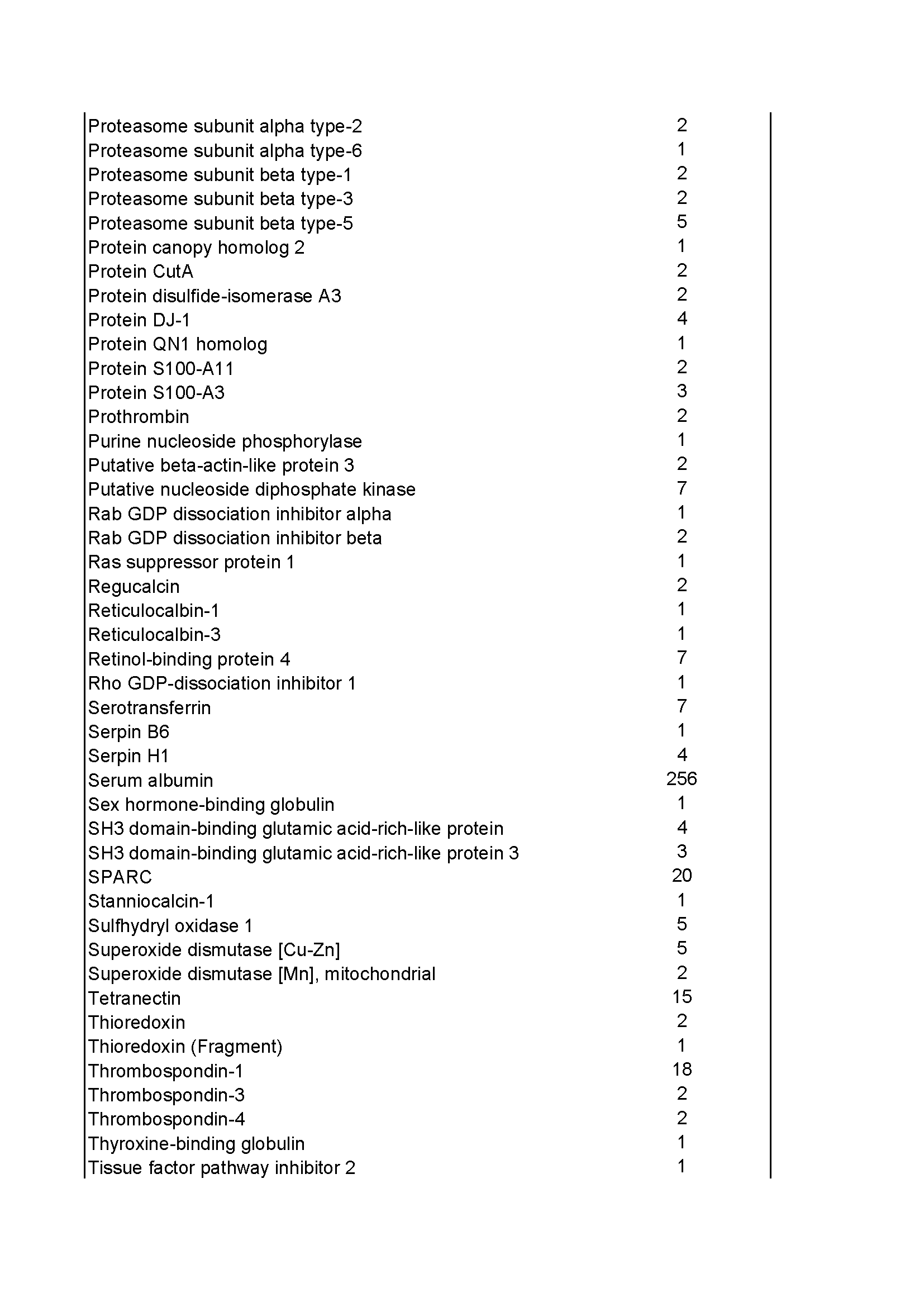

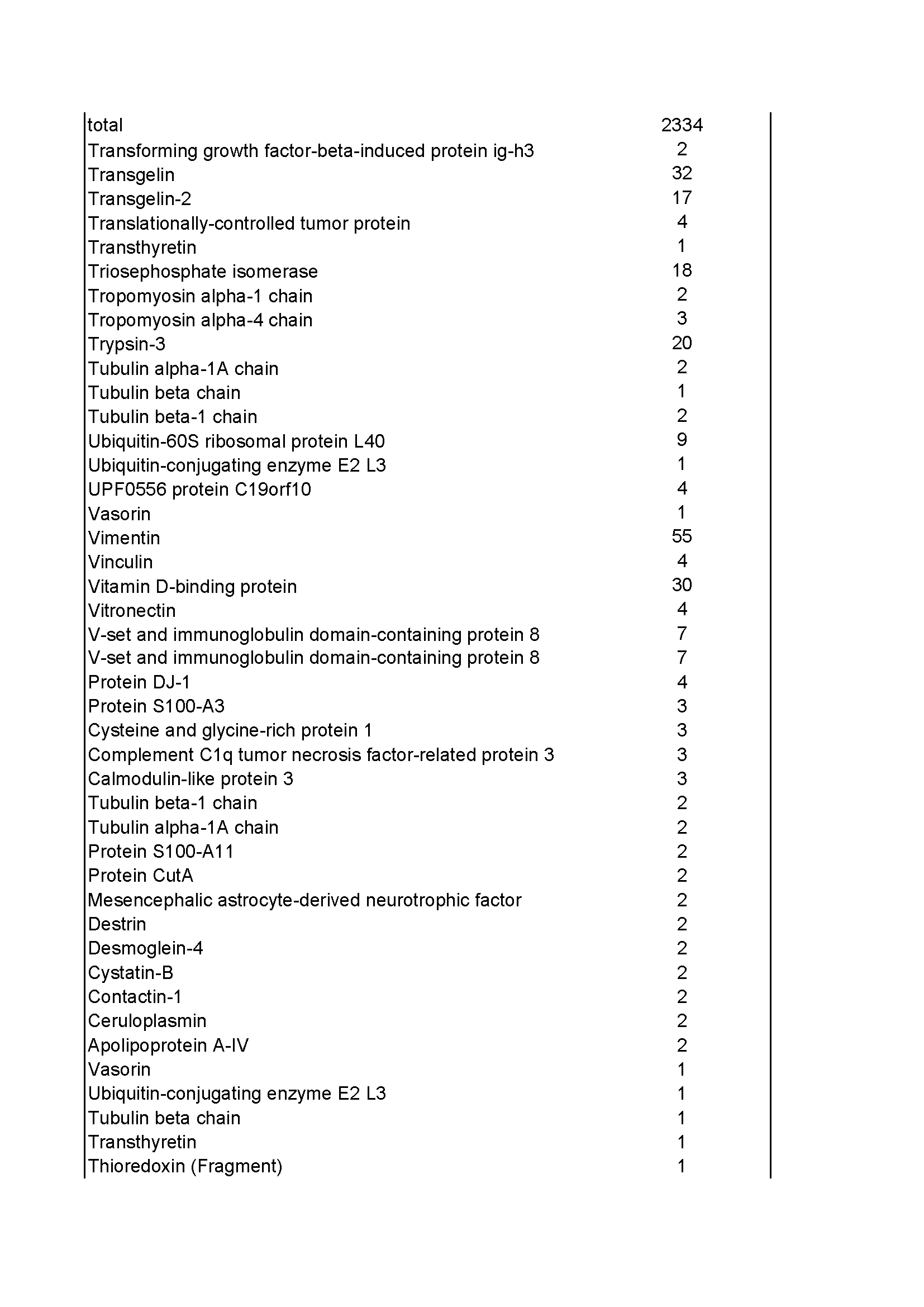

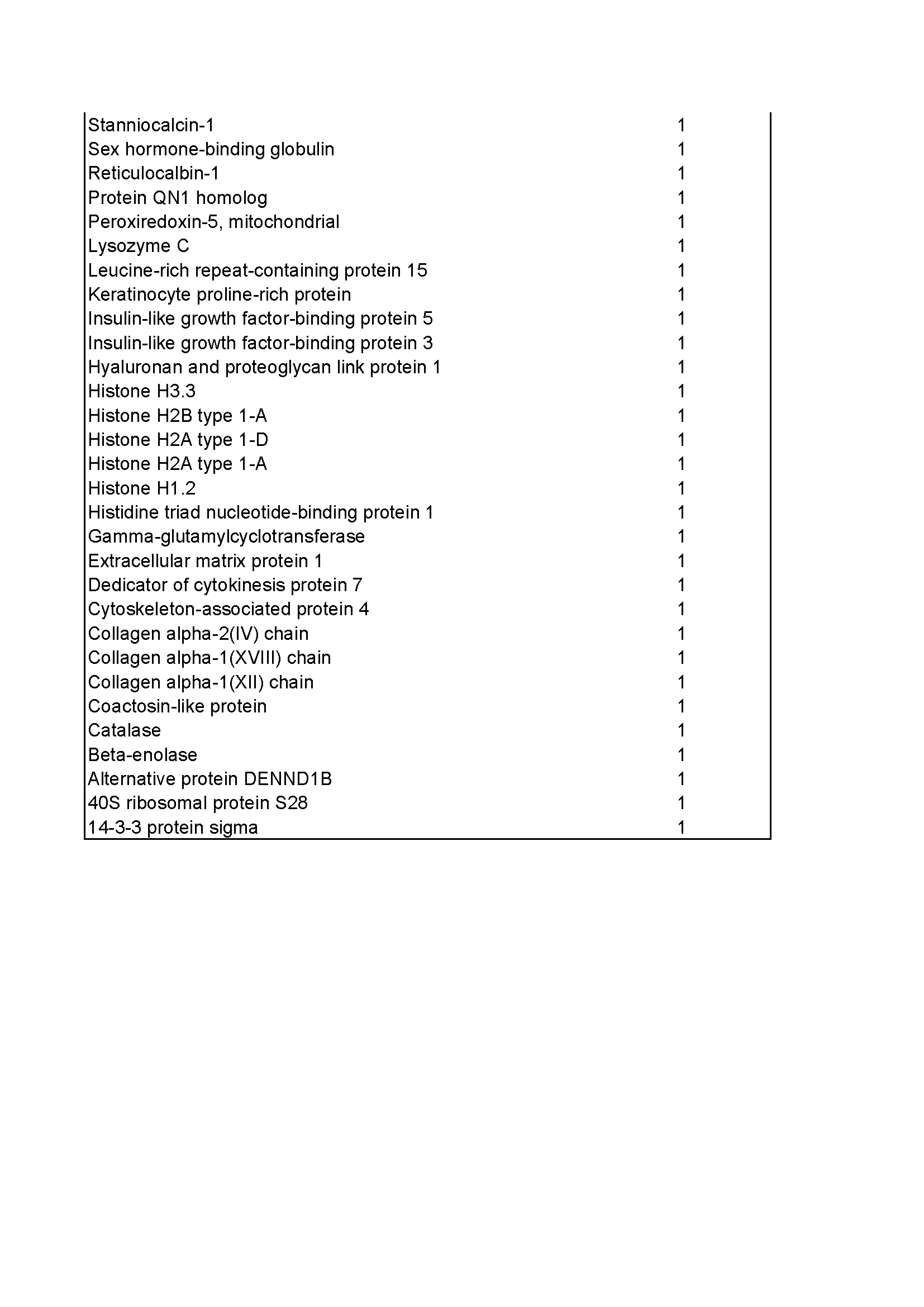
**
